# Current Status of PSMA-Targeted Agents Based on Small Molecules for Prostate Cancer in Preclinical Studies

**DOI:** 10.1007/s13139-025-00979-1

**Published:** 2026-01-26

**Authors:** Ayman Muzammal, Kyo Chul Lee, Sairan Eom

**Affiliations:** 1 Division of Applied RI, Korea Institute of Radiological and Medical Sciences (KIRAMS), 75 Nowon-Ro, Nowon-Gu, Seoul, 01812 Republic of Korea; 2 Radiological & Medical Sciences, University of Science and Technology (UST), Daejeon, Korea

**Keywords:** Prostate cancer, Prostate‐specific membrane antigen, PSMA-targeting small-molecules, Preclinical radioligand therapy

## Abstract

Prostate-specific membrane antigen (PSMA) has emerged as a clinically validated target for the diagnosis and treatment of prostate cancer using radioligand therapy (RLT). Among various PSMA-targeting ligands, urea-based small-molecules have gained limelight due to their favorable pharmacokinetic properties, high tumor penetration and convenient radiolabeling. Current preclinical research focuses on enhancing in vivo efficacy by optimizing ligand design, radionuclide selection, and improved pharmacokinetics. To reduce off-target toxicity in organs and enhance tumor accumulation, strategies involving the incorporation of albumin-binding moieties or linker modifications have been explored. Various studies have focused on PSMA-targeted therapies labeled with radionuclides, including β emitters (lutetium-177), α emitters (actinium-225 or astatine-211), and Auger electron emitters (iodine-125), that provide unique DNA damage mechanisms tailored to the tumor size and disease stage. This review summarizes recent approaches of PSMA-targeted small-molecules to optimize therapeutic efficacy while minimizing adverse effects on healthy tissues for RLT of prostate cancer.

## Introduction

Prostate cancer is the second most commonly diagnosed cancer in men worldwide, accounting for approximately 15% of all male cancer incidences and is responsible for a significant proportion of new cancer cases and cancer-related deaths in the male population [1, 2]. Early-stage prostate cancer can often be managed with local treatment modalities such as cancer radical prostatectomy or radiotherapy but clinical management becomes much more difficult in advanced stages, especially metastatic or castration-resistant prostate cancer (CRPC) [3]. Although therapeutic approaches including surgery, hormone therapy, and chemotherapy have been developed and applied to treat prostate cancer, however ongoing treatment for patients with advanced or metastatic prostate cancer are still limited and recurrences are frequent [4]. This suboptimal efficacy is primarily due to tumor heterogeneity, the emergence of resistance to androgen deprivation therapy and inadequate responses to systemic treatments [5, 6]. These limitations have led to increasing interest in developing targeted molecular therapies that can specifically select and bind to tumor-associated biomarkers to improve therapeutic efficacy and reduce systemic toxicity and damage to healthy tissues.

One such targeted approach is radioligand therapy (RLT), that combines tumor-specific ligands with therapeutic radionuclides to selectively deliver cytotoxic radiation to cancer cells while sparing normal tissues. By integrating the precision of molecular targeting with the therapeutic efficacy of radionuclide therapy, RLT has demonstrated promising outcomes in tumor control and reduction of systemic toxicity compared with conventional treatments [7]. In prostate cancer, RLT primarily targets prostate-specific membrane antigen (PSMA), a type-II transmembrane glycoprotein that has emerged as one of the most promising molecular targets for theranostic purpose. PSMA is extremely overexpressed on the surface of prostate cancer cells, particularly in high-stage and high-grade tumors, hormone resistance, metastatic diseases, and CRPC, where its expression is associated with tumor aggressiveness [8, 9]. Although recent literature has reported the salivary glands as dose-limiting organs in PSMA-targeted RLT, it is well known that PSMA is also physiologically expressed at low levels in normal tissues [10]. This restricted expression pattern between malignant and normal tissues provides the potential for highly specific therapeutic delivery while minimizing off-target toxicity such as salivary gland damage for the development of PSMA-targeted agents. Therefore, PSMA is currently regarded as one of the most promising targets for imaging and therapy in the field of nuclear medicine for prostate cancer [11].

Among various PSMA-targeted agents, small-molecule ligands have attracted considerable interest in both imaging and therapeutic applications due to their advantages over antibodies and nanoparticles, including low molecular weight, rapid blood clearance, deeper tumor penetration, and convenient radiolabeling [12, 13]. Most PSMA-targeted small molecules are based on a glutamate-urea-lysine (glu-urea-lys) scaffold, which mimics the natural substrate of PSMA and provides high affinity and specificity [14]. By conjugating these ligands with therapeutic radionuclides, such as β-emitters (lutetium-177), α-emitters (actinium-225, astatine-211), or Auger emitters, selective irradiation of PSMA-positive tumor tissues can be achieved while sparing surrounding normal cells [15, 16]. Although clinical trials have shown promising results, particularly with agents such as [^177^Lu]Lu-PSMA-617 [17], continued preclinical studies remain crucial. Preclinical studies not only facilitate the discovery and optimization of new ligand and radionuclide combinations, but also enable detailed pharmacokinetic analysis, toxicity profiling, and the exploration of combination therapies. In this review, we focus on recent advances and key roles of PSMA-targeted small-molecules in preclinical development to provide guidance for therapeutic application of prostate cancer from the viewpoint of nuclear medicine.

## Small-Molecule PSMA Ligands

**Fig. 1:**
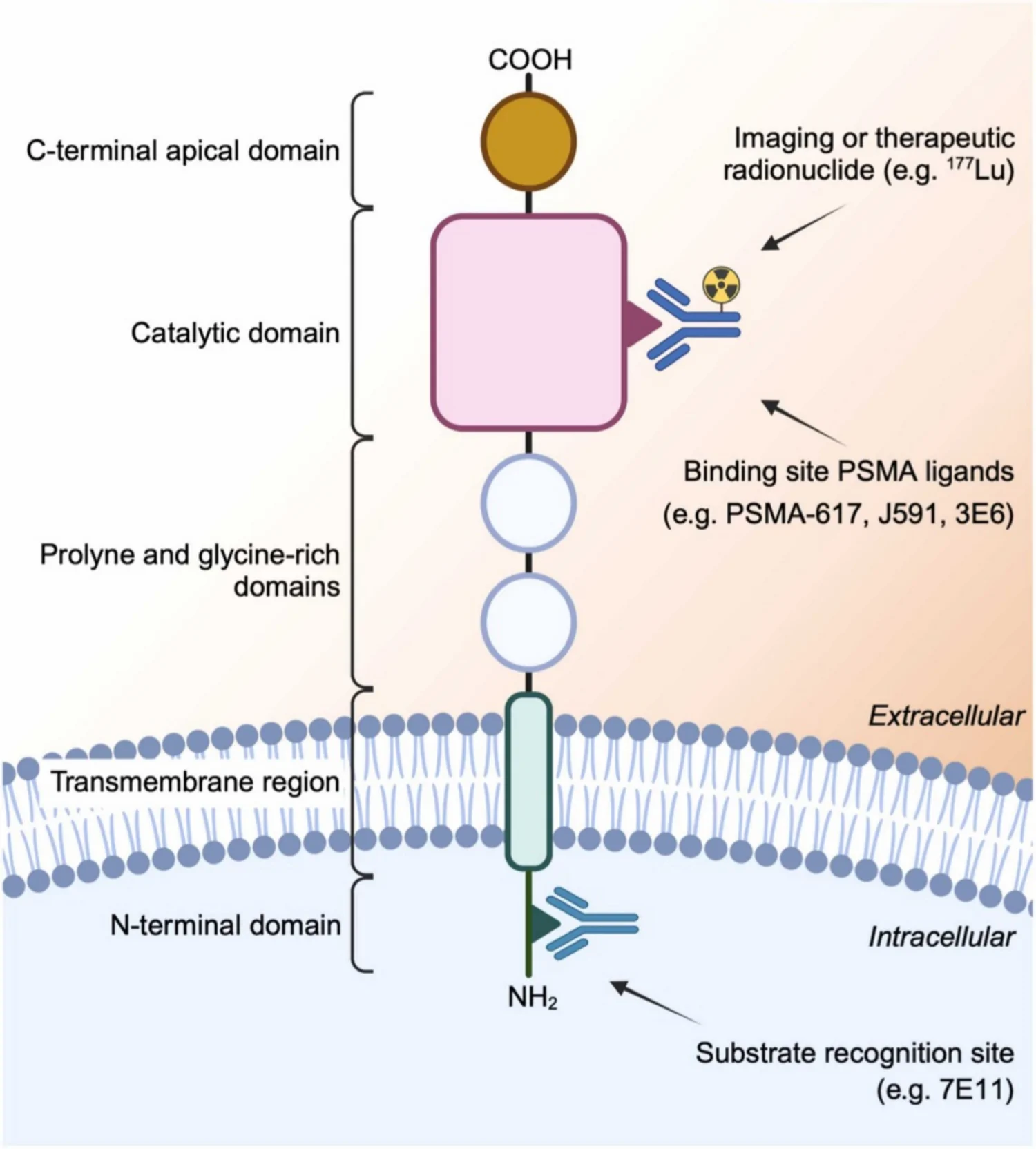
Molecular structure of PSMA highlighting its distinct structural domains and key ligand binding sites. It includes a short intracellular domain, a central transmembrane region, and a large extracellular portion. The extracellular region has two domains of unknown function connected by proline-rich and glycine-rich linkers, an important catalytic domain containing the substrate-binding site targeted by PSMA inhibitors such as antibodies or small molecules and a terminal domain of unknown function containing a helical dimerization motif. Adapted from Puik JR et al., Crit Rev Oncol Hematol. 2025;205:104556. Copyright © 2025 by the authors. Licensed under CC BY 4.0 [19]

**Fig. 2:**
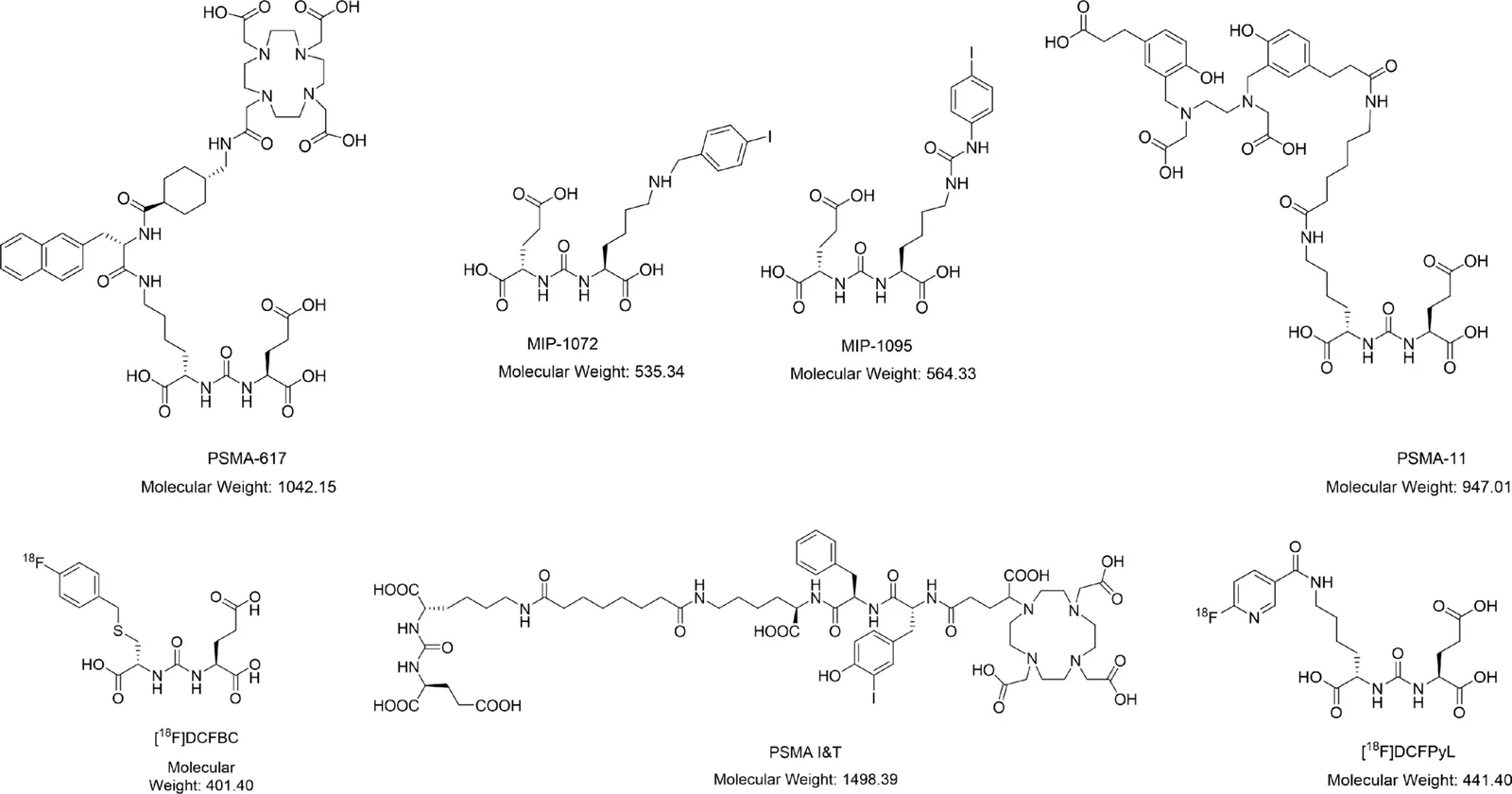
Chemical structure of important PSMA-ligands

In nuclear medicine, PSMA structure for targeting involves a PSMA binding domain, which is the part of the agent that recognizes and binds to the PSMA protein on cancer cells, and a domain to attach a therapeutic or imaging radionuclide [18]. This structure consists of a binding moiety (like small molecules or antibodies), a linker connecting the binder, and a radionuclide (Fig. 1) [19]. Anti-PSMA monoclonal antibodies, small molecule inhibitors, and PSMA-specific aptamers have been used as binding moieties for PSMA targeting [20]. PSMA-targeting antibodies offer high specificity to prostate cancer cells enabling accurate imaging and targeted therapy, but they also have drawbacks, including long circulation times that delay optimal imaging, the potential for nonspecific side effects and immunogenic response [21]. PSMA-specific aptamers, short DNA/RNA oligonucleotides, have attracted attention due to their high specificity and binding affinity with faster tissue penetration and tumor internalization and non-immunogenic nature as compared to antibodies. However, they are prone to nuclease degradation and structural modifications that can compromise binding affinity, and their small size also leads to much rapid renal clearance, thereby limiting circulation time and sufficient tumor accumulation [22]. On the other hand, small molecules have emerged most prominently, due to their high tumor penetration, faster blood clearance that minimizes non-specific binding and enhances tumor-to-background contrast, as well as the ease of radiolabeling, making them highly suitable for both imaging and therapeutic applications [23]. Small molecules for PSMA targeting have been extensively studied not only for their favorable pharmacokinetics but also for their diverse chemical structures, which influence binding affinity and radiolabeling strategies. In particular, PSMA-I&T and PSMA-617, labeled with lutetium-177 ([^177^Lu]Lu-PSMA-I&T and [^177^Lu]Lu-PSMA-617), are both representative small molecule PSMA-targeting radioligands used in targeted therapy of prostate cancer [24]. In addition, various radiolabeled small molecules (Fig. 2) like [^68^Ga]Ga-PSMA-11, [^18^F]DCFBC, [^18^F]DCFPyL, [^123^I]MIP-1072 and [^123^I]MIP-1095 have shown considerable efficacy and widespread use for molecular imaging [25–28].

**Fig. 3:**
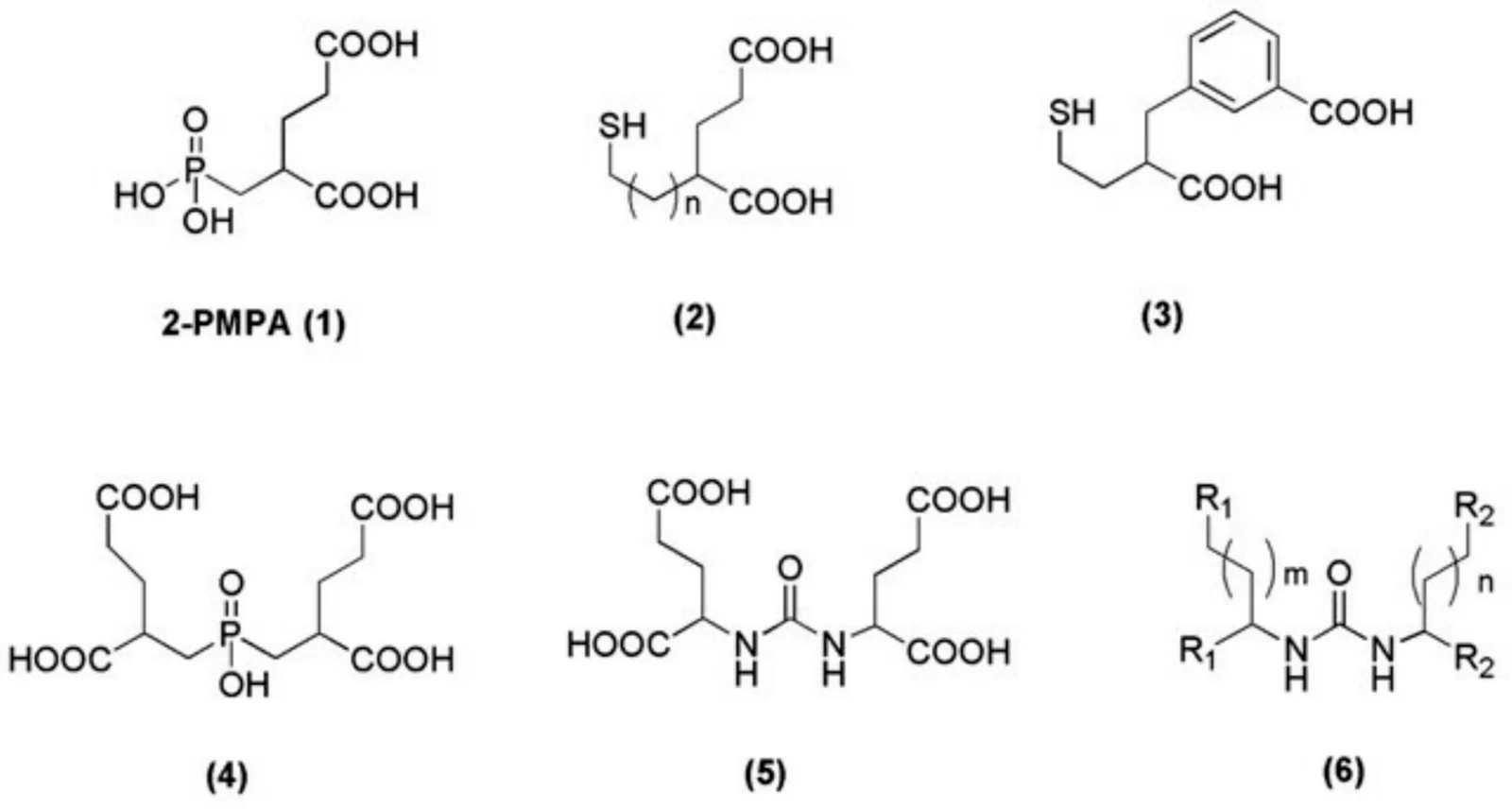
Representative chemical structures of PSMA-targeting small molecule inhibitors. Phosphorus-based compound (1) 2-PMPA, and compound (4), thiol-based compound (2) and (3), and urea-based compounds (5) and (6). Adapted from Gourni E, et al., Molecules. 2017;22:523. Copyright © 2017 by the authors. Licensed under CC BY 4.0 [29]

PSMA-targeting small molecule inhibitors are chemically classified into three main groups: phosphorus-based, thiol-based, and urea-based compounds (Fig. 3) [29]. Before urea-based ligands emerged as main strategy for targeting PSMA due to their superior binding affinity, significant efforts were made for the development of phosphorus-based PSMA inhibitors such as 2-PMPA, GPI 5232 and GPI 18431 as well as thiol-based PSMA ligands such as 2-MPPA and E2072. Phosphorus-based PSMA inhibitors face two main challenges; in vivo competition with endogenous phosphates and a reversible internalization profile. However, thiol-based PSMA ligands are susceptible to oxidation, which can compromise their metabolic stability and trigger undesirable immune responses [30]. Among these three, urea-based small molecules, consist of a glu-urea-lys binding motif, a linker, and a radiolabeling moiety, demonstrate the highest binding affinity for PSMA and serve as the primary scaffold in the development of novel ligands [14]. The glu-urea-lys pharmacophore has been covalently functionalized with different chelators, such as DOTA (1,4,7,10-tetraaza-cyclododecane-1,4,7,10-tetraacetic acid), DOTAGA (1,4,7,10-tetraazacyclododecane,1-(glutaric acid)−4,7,10-triacetic acid), NOTA (1,4,7-triaza-cyclononane-1,4,7-triacetic acid) and HBED-CC (N,N′-bis[2-hydroxy-5-(carboxyethyl)benzyl]ethylenediamine-N,N′-diacetic acid) for radiometals labeling. These macrocyclic bifunctional chelators are particularly advantageous because they enable efficient and stable radiolabeling with radiometals (gallium-68 and lutetium-177) [31].

Beyond the choice of chelator, other structural components, particularly the linker and the radiolabeling moiety also play a pivotal role in determining the pharmacokinetic and pharmacodynamic behavior of PSMA-targeting small molecules [32]. A rational linker design can significantly improve tumor specificity and pharmacokinetics by reducing non-specific organ uptake and enhancing tumor-to-background contrast [33]. The choice of radionuclide also influences the therapeutic outcome of PSMA-targeted agents by determining the type and range of radiations emitted, which affects tumor cell killing efficacy, tissue penetration, and off-target toxicity [34]. Structural modifications in these components directly impact safety profiles by reducing radioligand accumulation in susceptible organs like kidneys and salivary glands, which are common dose-limiting sites during PSMA-targeted therapy [10]. These structure-functional relationships have led to the rational development of novel radiolabeled PSMA-targeted ligands, which are now being investigated for their optimized therapeutic performance, safety and radiolabeling versatility.

## Preclinical Studies of PSMA-targeted Agents

For RLT of prostate cancer, PSMA-targeted small molecules are currently be labeled with various therapeutic radionuclides, including beta (β), alpha (α), and Auger electron emitters, each offering distinct properties that influence their therapeutic efficacy, tissue penetration, and off-target toxicity profiles as summarized in Table 1 [35, 36]. Based on their distinct physical and biological characteristics, the choice of radionuclide in PSMA-targeted therapy depends on tumor size, location, and treatment goal. β-emitters possess a lower linear energy transfer (LET) (~ 0.2 keV/μm), leading predominantly to the induction of DNA single-strand breaks (SSBs) as the primary mechanism of cytotoxicity. Their relatively longer tissue penetration (0.05–12 mm) enables a crossfire effect, enhancing dose uniformity within the tumor mass and making them more suitable for treating medium to large tumors [37]. On the other hand, α-emitters are ideal for small-volume disease or micrometastases due to their high LET (80 keV/μm) and short path length (40–100 μm), which induce dense ionization leading to irreparable DNA double-strand breaks (DSBs), thereby enabling potent cytotoxicity with minimal impact on surrounding healthy tissue [38]. Although α-radiation is highly effective in damaging tumor cells, its clinical use is limited by off-target toxicity to normal tissues expressing PSMA, such as the salivary glands, thereby restricting the maximum safe dose [39]. Auger electron emitters, characterized by extremely short range (2–500 nm) and moderate-to-high LET (4–26 keV/µm), require precise intracellular localization to achieve therapeutic efficacy, making them particularly suitable for targeting single cells or microscopic lesions when delivered with highly specific carrier molecules [40, 41]. Due to the distinct therapeutic characteristics of radionuclides as compared in Table 1, extensive research has been conducted to identify optimal small molecules and radioisotopes capable of effectively targeting PSMA for the treatment of prostate cancer. In this session, recent advances of PSMA-targeted agents for RLT of prostate cancer in preclinical studies are investigated as shown in Table 2.

**Table 1:** Comparison of physical and biological characteristics of α, β particles, and Auger electron

Radionuclide	Energy	Range	LET^1)^	DNA damage
Beta (β)	50–230 keV	≤ 12 mm	~ 0.2 keV/μm	SSBs^2)^
Alpha (α)	4–9 MeV	40–100 μm	80 keV/μm	DSBs^3)^
Auger electron	25–80 keV	2–500 nm	4–26 keV/μm	DSBs

1) LET: Linear Energy Transfer, 2) SSBs: Singal Strand Breaks, 3) DSBs: Double Strand Breaks

**Table 2:** Comparison of Alpha, Beta, and Auger-labeled Urea based PSMA small-molecules

Type of radionuclide	Radionuclide	Small molecule	Cell line	In vitro assay	Tumor uptake (%ID/g) at 24 h	T/K ratio at 24 h	Ref
Beta-emitter	Lutetium-177	HTK01169	LNCaP	Competition Binding, K_i_ = 0.04 nM	55.9	0.45	[42]
	Lutetium-177	HTK03121	LNCaP	Competition Binding, K_i_ = 2.40 nM	104	3.06	[43]
	Lutetium-177	HTK03123	LNCaP	Competition Binding, K_i_ = 1.76 nM	70.8	3.66	[43]
	Lutetium-177	PSMA-ALB 02	PC3-PIP	Uptake = 60% and Internalization = 26% cell binding at 4 h	76.4	7.16	[44]
	Lutetium-177	PSMA-ALB-05	PC3-PIP	Uptake = 60% and Internalization = 20% cell binding at 4 h	79.4	3.38	[44]
	Lutetium-177	PSMA-ALB-07	PC3-PIP	Uptake = 63% and Internalization = 22% cell binding at 4 h	84.6	1.64	[44]
	Lutetium-177	PSMA-ALB-53	PC3-PIP	Uptake = 59% and Internalization = 25% cell binding at4 h	53.8	2.17	[45]
	Lutetium-177	PSMA-ALB-56	PC3-PIP	Uptake = 55% and Internalization = 17% cell binding at 4 h	103.9	10.8	[45]
	Lutetium-177	RPS-061	LNCaP	IC_50_ = 4.1 nM	18.7	0.45	[46]
	Lutetium-177	RPS-063	LNCaP	IC_50_ = 1.5 nM	21.0	0.40	[46]
	Lutetium-177	RPS-066	LNCaP	IC_50_ = 5.2 nM	11.9	1.92	[46]
	Lutetium-177	RPS-067	LNCaP	IC_50_ = 9.5 nM	5.7	1.25	[46]
	Lutetium-177	RPS-068	LNCaP	IC_50_ = 2.1 nM	26.8	0.30	[46]
	Lutetium-177	RPS-069	LNCaP	IC_50_ = 3.8 nM	12.9	0.95	[46]
	Lutetium-177	RPS-071	LNCaP	IC_50_ = 10.8 nM	~ 7.5	3.2	[47]
	Lutetium-177	RPS-072	LNCaP	IC_50_ = 6.7 nM	34.9	4.7	[47]
	Lutetium-177	RPS-077	LNCaP	IC_50_ = 1.7 nM	27.4	~ 0.9	[47]
	Iodine-131	RPS-027	LNCaP	IC_50_ = 15 nM	8.13	~ 3	[48]
	Copper-64	DOTHA_2_-PSMA	LNCaP	IC_50_ = 64 nM, Uptake = 34.5 and Internalization = 34.1% IA/10^6^ cells over 48 h	23.8 (%ID/cc)	N/A	[49]
Alpha-emitter	Lead-212	CA012	C4-2	Competition Binding, K_i_ = 24.3 nM and Internalization = 15.6%IA/10^6^ cells	7.81	4.85	[50]
	Lead-212	NG001	C4-2	Binding affinity, K_d_ = 22.0 ± 4.1 nM and internalization = 21.0%IA/10^6^ cells	13.6	1.41	[51, 52]
	Actinium-225	L1	PC3-PIP	Uptake, Clonogenic survival (N/A)	49.0	32.6	[53]
	Bismuth-213	L1	PC3-PIP	Uptake, Clonogenic survival (N/A)	N/A	N/A	[53]
	Astatine-211	3-Lu	PC3-PIP	Uptake = 13.4% IA and Internalization = 27.7% cell bound	9.46	130	[54]
	Astatine-211	PSMA-1	LNCaP	Uptake = ~ 22% uptake/mg protein at 30 min	8.7	N/A	[55]
	Astatine-211	PSMA-5	LNCaP	Uptake = ~ 43% uptake/mg protein at 30 min	40.7	N/A	[55]
	Astatine-211	PSMA-6	LNCaP	Uptake (data N/A)	18.1	N/A	[55]
	Astatine-211	NpG-PSMA	PC3-PIP	Uptake = ~ 54% uptake/mg at 4 h	41.08	62.2	[56]
Auger-emitter	Iodine-125	DCIBzL	PC3-PIP	Competition binding K_i_ = 0.01 nM, Subcellular localization = 14% in perinuclear area	N/A	N/A	[57]
	Cobalt-58 m	DOTA-PSMA-617	PC3-PIP	Subcellular localization of PC3-PIP = 9.7% IA/10^6^ and LNcaP = 12.6% IA/10^6^ at 4 h in nucleus	6.2	7.0	[58]
	Cerium-134	PSMA-617	PC3-PIP	Uptake = 89.6% and Internalization = 34.8% cell binding at 4 h	4.5	7.3	[59]

**K**_i_ = Inhibition constant, **K**_d_ = Dissociation Constant, **IC**_₅₀_ = Half maximal inhibitory concentration, **IA**= Incubated Activity, **ID/g** = Injected Dose per gram, **T/K** = Tumor-to-Kidney ratio, ~ indicate the approximate value

### Beta Emitter Labeled PSMA-Targeted Agents

For prostate cancer RLT, β-emitters such as lutetium-177, iodine-131, yttrium-90, copper-67 and terbium-161 have been investigated by labeling with PSMA-targeted small molecules [60]. Among the various β-emitters labeled PSMA-targeted agents, [^177^Lu]Lu-PSMA-617 (Fluvicto) has emerged as a representative treatment for mCRPC, receiving FDA approval in 2022 [61, 62]. This approval was based on multiple clinical studies demonstrating its favorable safety profile and significant therapeutic efficacy, including high rates of prostate specific antigen (PSA) decline and manageable adverse events. Notably, [^177^Lu]Lu-PSMA-617 showed superiority over conventional therapies in delaying disease progression, as confirmed in major clinical trials like the TheraP and VISION studies, which compared this treatment to other standard options [63–68].

**Fig. 4:**
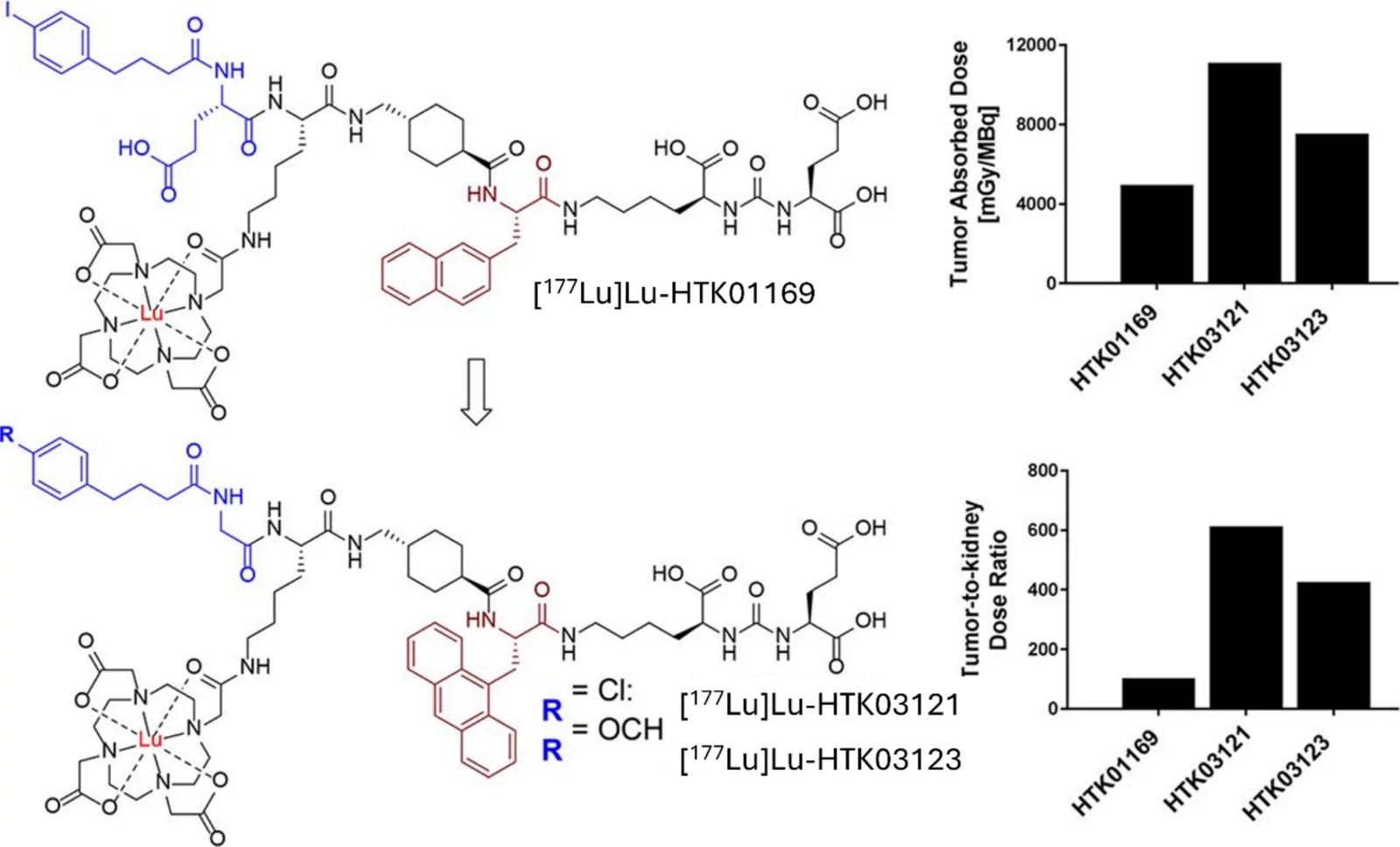
Structural optimization of HTK01169 leading to HTK03121 and HTK03123 to enhance tumor-to-kidney absorbed dose ratio. This research was originally published in JNM. Kuo HT, et al. ^177^Lu-Labeled Albumin-Binder-Conjugated PSMA-Targeting Agents with Extremely High Tumor Uptake and Enhanced Tumor-to-Kidney Absorbed Dose Ratio. J Nucl Med. 2021;62:521–7. © SNMMI [43]

Although, [^177^Lu]Lu-PSMA-617 shows favorable therapeutic efficacy, its associated toxicities such as xerostomia and hematologic adverse events have prompted ongoing preclinical efforts to optimize its structure for enhanced tumor targeting and reduced off-target effects [68]. A major limitation of current PSMA-targeting radioligands is their relatively rapid blood clearance, which can compromise tumor uptake and reduce therapeutic efficacy. To address this, the researchers have focused on structural modifications particularly the incorporation of albumin-binding moiety (ABM) to prolong their circulation time and enhance tumor accumulation [69]. For example, [^177^Lu]Lu-HTK01169, a close derivative of PSMA-617 incorporating a highly lipophilic ABM (N-[4-(p-iodophenyl)butanoyl]-Glu) had enabled strong reversible binding to serum albumin, thereby reducing the fraction of unbound radioligand in plasma (< 1% vs ~ 17% for PSMA-617). It also showed enhanced PSMA affinity (K_i_ = 0.04 ± 0.01 nM), representing a six-fold improvement over [^177^Lu]Lu-PSMA-617 (K_i_ = 0.24 ± 0.06 nM). This translated to enhanced peak tumor uptake (55.9 ± 12.5%ID/g) in LNCaP xenografts, a 3.7-fold increase relative to [^177^Lu]Lu-PSMA-617 (15.1 ± 5.58%ID/g). Dosimetry analysis revealed an 8.3-fold higher tumor absorbed dose, enabling a 4-fold reduction in administered activity to achieve an equivalent median survival (4.6 MBq vs. 18.5 MBq). However, the improved tumor dose was accompanied by a substantial increase in normal organ exposure, most notably a 17.1-fold higher kidney radiation dose [42]. Following HTK01169, subsequent analogues HTK03121 and HTK03123 (Fig. 4) were developed by combining the tranexamic acid-9-anthrylalanine (affinity-modifying group) with N-(4-(p-chlorophenyl)butanoyl)-Gly and N-(4-(p-methoxyphenyl)butanoyl)-Gly (as ABM) respectively. By replacing the Glu in the N-(4-(p-iodophenyl)butanoyl)-Glu group with Gly removed the free carboxylate responsible for excessive kidney uptake, while substituting the highly lipophilic iodo group (LogP = 3.39) with less lipophilic chloro and methoxy groups (LogP = 2.59 and 2.08 respectively) accelerated renal clearance, together improving tumor-to-kidney dose ratios. This dual optimization also enhanced tumor uptake; 86% increase for [^177^Lu]Lu-HTK03121 and 23% for [^177^Lu]Lu-HTK03123 compared to [^177^Lu]Lu-HTK01169, with [^177^Lu]Lu-HTK03121 achieving peak tumor uptake values as high as 104%ID/g. The kidney uptake was significantly reduced, yielding approximately 62% lower renal absorbed dose relative to [^177^Lu]Lu-HTK01169. As a result, these analogues achieved 6.0- and 4.1-fold increase in tumor-to-kidney absorbed dose ratios compared to [^177^Lu]Lu-HTK01169, and significantly outperformed [^177^Lu]Lu-PSMA-617 with 2.9- and 2.0-fold higher ratios, respectively. Enhanced blood retention was modest, consistent with albumin binding, but no significant marrow dose concerns were observed [43].

**Fig. 5:**
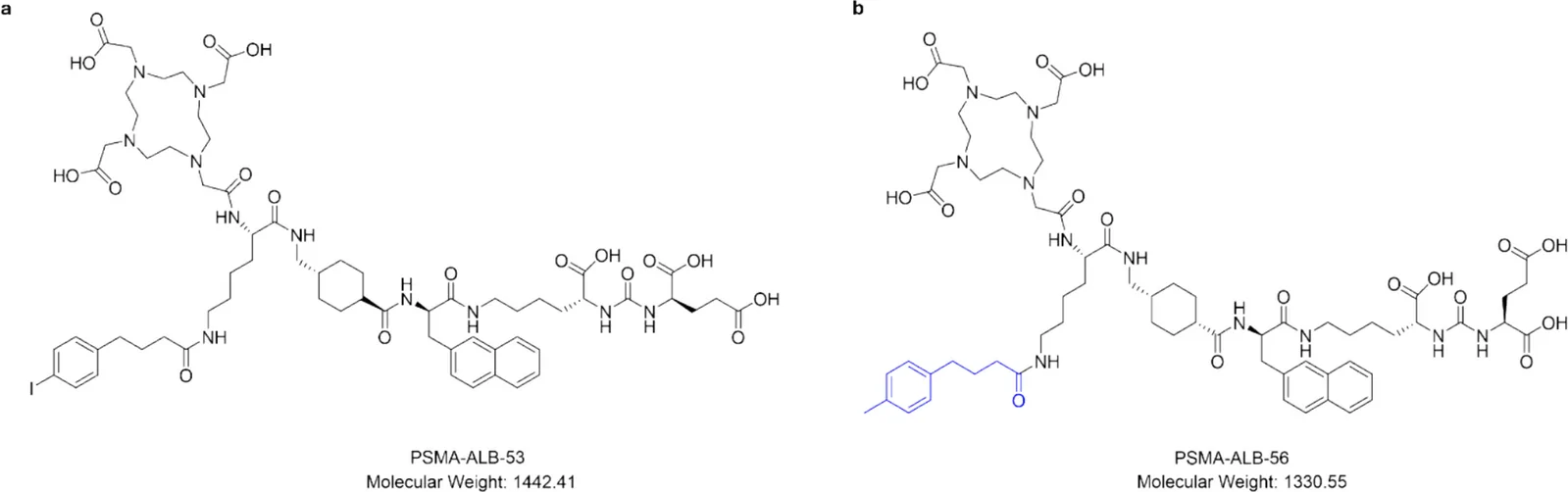
Chemical structure of (**a**) PSMA-ALB-53 and (**b**) PSMA-ALB-56

In parallel, other urea-based ligands, PSMA-ALB-02, PSMA-ALB-05, and PSMA-ALB-07 incorporating 4-(p-iodophenyl) butyric acid as the ABM have been developed to enhance tumor targeting and pharmacokinetics. These ligands differ in linker composition, with [^177^Lu]Lu-PSMA-ALB-05 and [^177^Lu]Lu-PSMA-ALB-07 containing additional D-aspartate residues aimed at modulating hydrophilicity and renal clearance. Tumor uptake at 24 h post-injection was highest for [^177^Lu]Lu-PSMA-ALB-07 (84.6 ± 14.2%ID/g) followed by [^177^Lu]Lu-PSMA-ALB-05 (79.4 ± 11.1%ID/g) and [^177^Lu]Lu-PSMA-ALB-02 (76.4 ± 2.5%ID/g). However, kidney retention varied substantially: [^177^Lu]Lu-PSMA-ALB-02 showed the lowest renal uptake (10.7 ± 0.92%ID/g), whereas [^177^Lu]Lu-PSMA-ALB-05 and [^177^Lu]Lu-PSMA-ALB-07 showed elevated kidney accumulation (23.9 ± 4.02 and 51.9 ± 6.34%ID/g respectively), raising concerns about potential nephrotoxicity [44]. Further optimization of ABM and linkers resulted in two novel compounds, both combine a PSMA-binding motif and a DOTA chelator with distinct ABM: a 4-(p-iodophenyl)butyric acid in PSMA-ALB-53 and a p-(tolyl)butyric acid in PSMA-ALB-56 (Fig. 5). Tumor uptake in PSMA-positive PC-3 PIP xenografts was similar for both [^177^Lu]Lu-PSMA-ALB-53 and [^177^Lu]Lu-PSMA-ALB-56. However, [^177^Lu]Lu-PSMA-ALB-53 showed 15-fold higher plasma protein binding and prolonged blood retention due to the strong albumin-binding affinity of the 4-(p-iodophenyl) moiety compared to the p-(tolyl) moiety in [^177^Lu]Lu-PSMA-ALB-56, resulting in a 5-fold lower tumor-to-blood ratio and potentially higher off-target exposure. In contrast, [^177^Lu]Lu-PSMA-ALB-56 cleared faster through the kidneys due to reduced serum albumin binding, yielding a 3-fold higher tumor-to-kidney ratio and reduced nephrotoxicity risk. Compared to [^177^Lu]Lu-PSMA-ALB-02, [^177^Lu]Lu-PSMA-ALB-56 increased tumor uptake by 25% and lowered kidney retention by 30%. In therapeutic evaluations, [^177^Lu]Lu-PSMA-ALB-56 also outperformed [^177^Lu]Lu-PSMA-617 at equal doses, significantly delaying tumor growth and extending survival, with no observed toxicity [45].

**Fig. 6:**
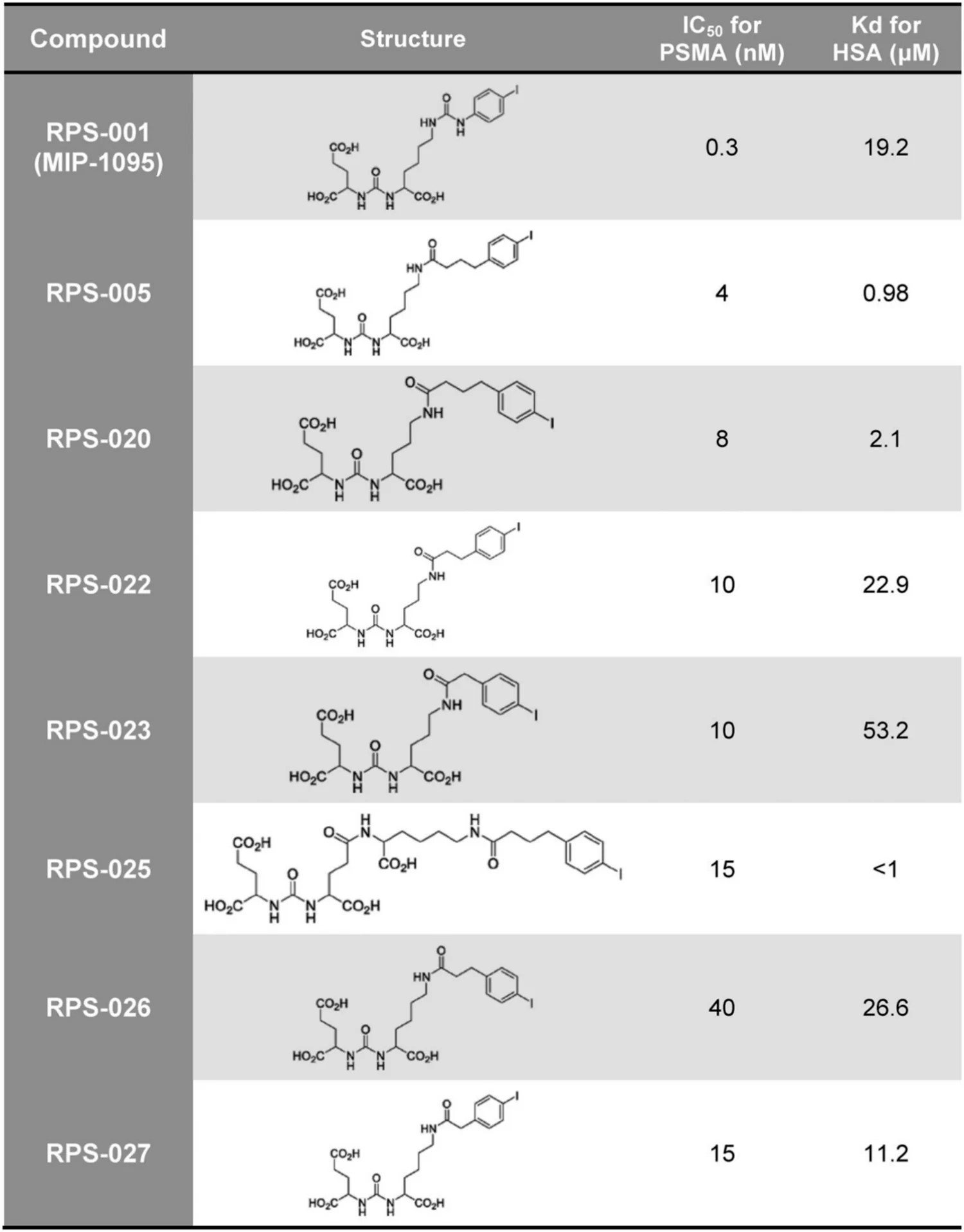
Chemical structure of RPS series compounds and their affinities for PSMA and HAS. This research was originally published in JNM. Kelly JM, et al. Dual-Target Binding Ligands with Modulated Pharmacokinetics for Endoradiotherapy of Prostate Cancer. J Nucl Med. 2017;58:1442–9. © SNMMI. [48]

**Fig. 7:**
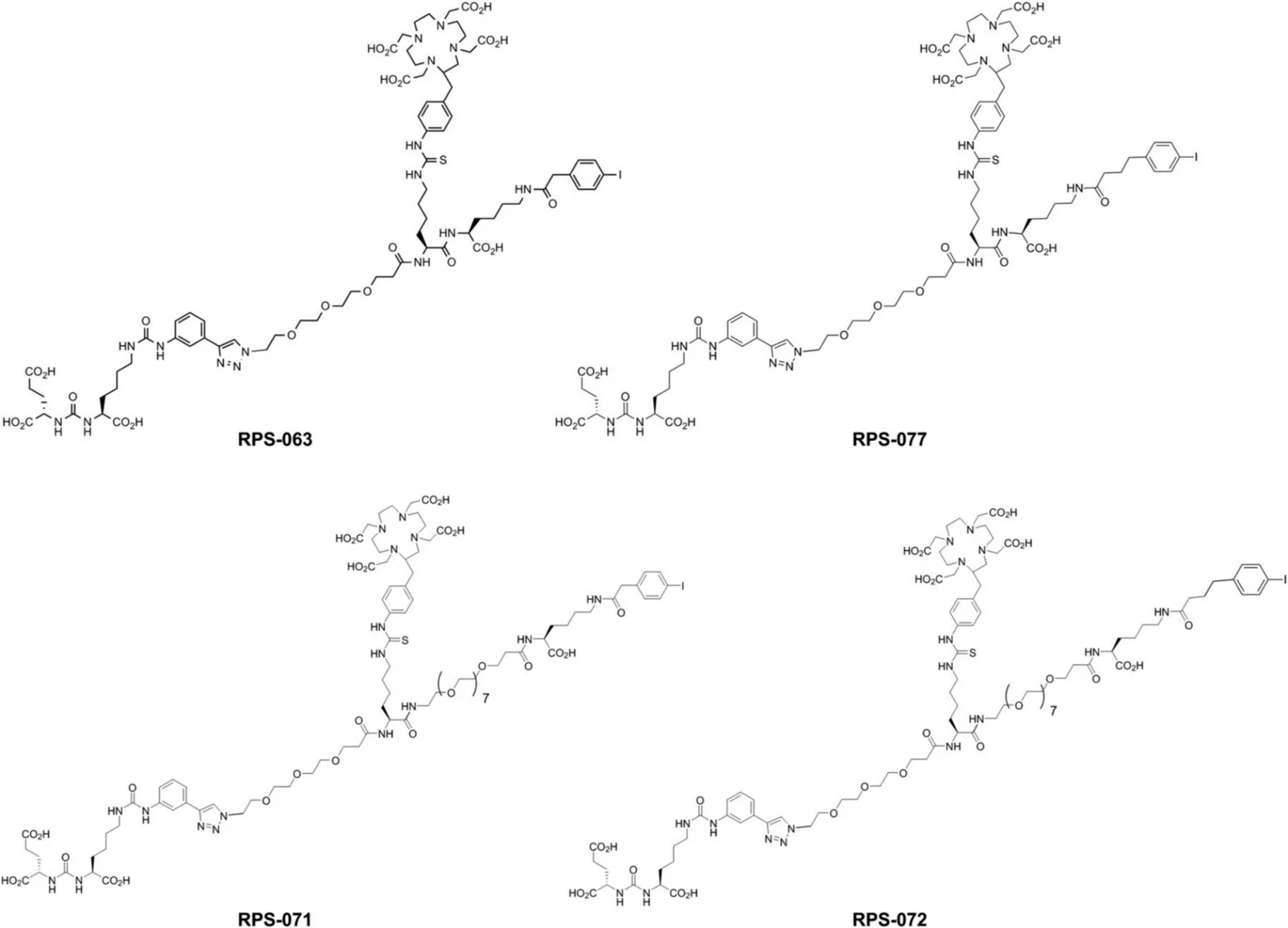
Chemical structure of four structurally related trifunctional PSMA-targeting small molecules. This research was originally published in JNM. Kelly JM, et al. Albumin-Binding PSMA Ligands: Implications for Expanding the Therapeutic Window. J Nucl Med. 2019;60:656–63. © SNMMI [47]

Another innovative approach focused on designing dual-target binding ligands capable of simultaneously binding PSMA and human serum albumin (HSA) for modulating pharmacokinetics. A series of novel urea-based ligands (RPS-001, RPS-005, RPS-020, RPS-022, RPS-023, RPS-025, RPS-026, and RPS-027 (Fig. 6) with varying affinities for PSMA and HSA, were labeled with iodine-131. Among all, [^131^I]RPS-027 showed balanced kinetics (IC₅₀ = 1–10 nM and K_d_ = 11 μM), with highest tumor uptake (12.41%ID/g) at 3 h, kidney uptake of 15.21%ID/g at 12 h reducing to 1.0%ID/g at 48 h and tumor-to-kidney ratios > 2 by 18 h. It also exhibited strong and prolonged tumor uptake, efficient clearance from blood within 48 h, and lower kidney accumulation compared to MIP-1095 [48]. Limitations in radionuclide choice and binding optimization of RPS-027 laid the foundation of a new class of trifunctional ligands (RPS-061, RPS-063, RPS-066, RPS-067, RPS-068 and RPS-069), combining PSMA-binding moiety (triazolylphenylurea), an ABM (N^ε^-(2-(4-iodophenyl)acetyl)lysine) and a bifunctional chelator (p-SCN-Bn-DOTA) linked via polyethylene glycol (PEG) chains of varying lengths (PEG4, PEG3, PEG8, PEG12, PEG0, and PEG6, respectively) compatible with various radiometals (gallium-68, lutetium-177, actinium-225). PSMA affinity remained high (IC₅₀ = 1–10 nM) across all ligands exhibiting an inverse correlation with linker length. Among all ligands, [^177^Lu]Lu-RPS-063 (IC₅₀ = 1.5 ± 0.3 nM) showed the highest tumor uptake (30.0 ± 6.9%ID/g) exceeding that of [^177^Lu]Lu-PSMA-617, although kidney uptake remained a concern [46]. Following this, RPS-071, RPS-072 and RPS-077 (Fig. 7) were developed, all featuring the common structural scaffold of RPS-063 comprising PSMA-targeting moiety linked to DOTA via a PEG3 spacer. RPS-063 and RPS-071 contained N^ε^-(2-(4-iodophenyl)acetyl)lysine, while RPS-072 and RPS-077 incorporate 4-(4-iodophenyl)butanoic acid as the ABM. In RPS-063 and RPS-077, the ABM was directly coupled to the core through a peptide bond, while in RPS-071 and RPS-072, the ABM was separated by an additional PEG8 linker. These structural variations had minimal impact on PSMA affinity but significantly influenced albumin binding and pharmacokinetics. In LNCaP xenograft models, [^177^Lu]Lu-RPS-072 (IC₅₀ = 6.7 ± 3.7 nM) showed highest tumor uptake (34.9 ± 2.4%ID/g) at 24 h and a 1.5-fold greater tumor AUC than [^177^Lu]Lu-RPS-063, along with a superior tumor-to-kidney AUC ratio (4.7 ± 0.3 vs 0.4 ± 0.02 respectively) due to stronger ABM. The extended PEG linker (in RPS-071 and RPS-072) modestly enhanced blood clearance and markedly accelerated kidney clearance without impacting tumor retention. Overall, fine-tuning of the ABG and PEG linker length effectively optimized albumin binding, resulting in improved tumor retention and better therapeutic efficacy with reduced off-target toxicity [47].

**Fig. 8:**
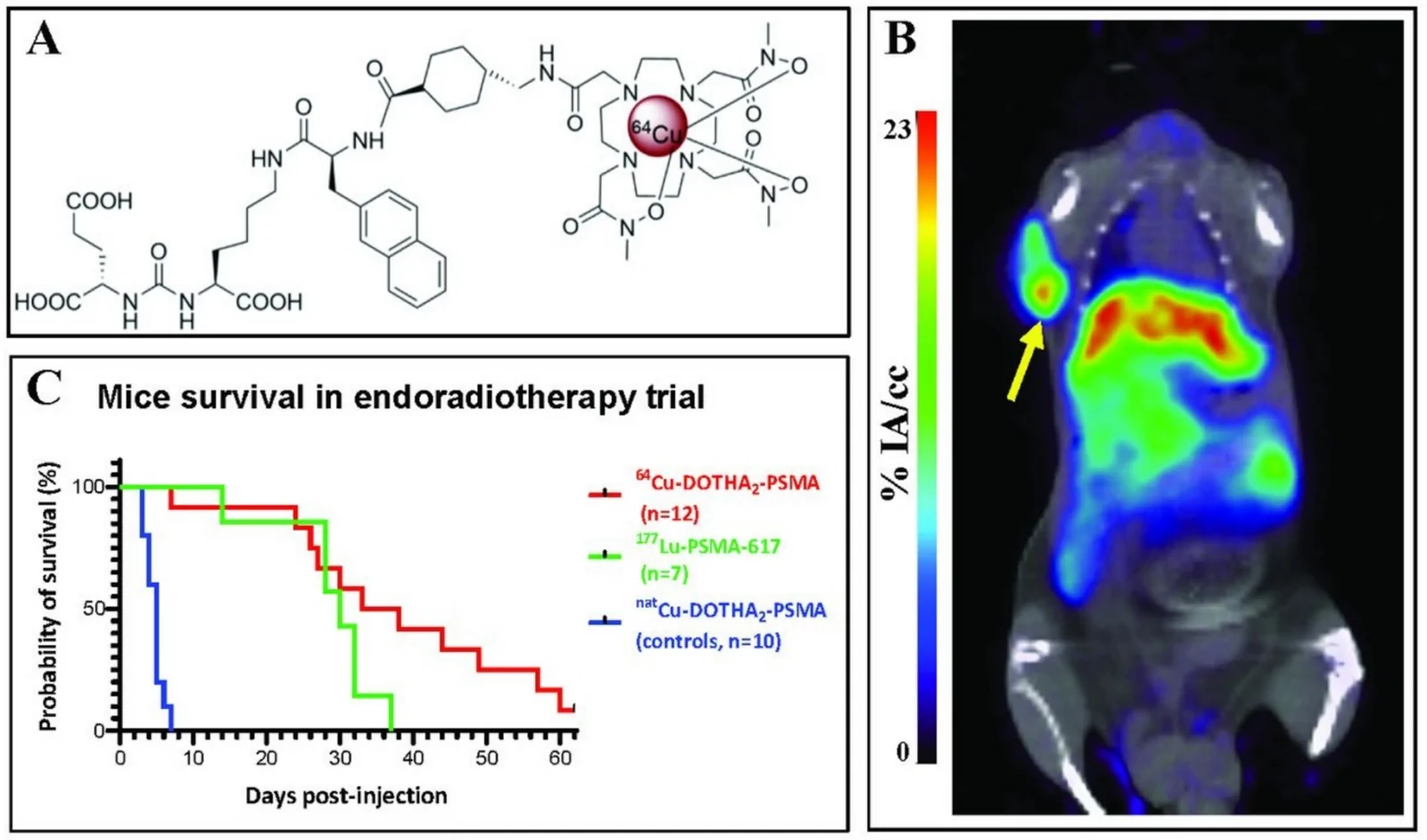
Chemical structure of theranostic agent [^64^Cu]Cu-DOTHA_2_-PSMA (**a**). PETC/CT image of LNCaP tumor-bearing NRG mice at 4 h post injection (**b**). Probability of survival (%) in comparison to non-radiactive control and [^177^Lu]Lu-PSMA-617 (**c**). This research was originally published in JNM. Milot M-C, et al. The journey to a novel theranostic for prostate cancer: the preclinical study of.^64^Cu-DOTHA_2_-PSMA. J Nucl Med. 2022;63:4025. © SNMMI [49]

Meanwhile, therapeutic PSMA radioligands often lack suitability for PET imaging, which is critical for patient selection and treatment monitoring, so they are typically paired with diagnostic PSMA agents; commonly exemplified by the [^68^ Ga]Ga/[^177^Lu]Lu-PSMA-617 theranostic pair. However, discrepancies between imaging-based PSMA expression and therapeutic response have been observed in some cases, resulting in suboptimal clinical outcomes. To overcome these limitations, [^64^Cu]Cu-DOTHA_2_-PSMA was evaluated preclinically as a novel theranostic agent for prostate cancer because copper-64 allows for simultaneous or sequential use for both imaging (using the β^+^ emissions for PET scans) and therapy (using the β^−^ and Auger electrons). The compound demonstrated strong PSMA-targeting characteristics, including high binding affinity (IC_50_ = 64 ± 50 nM) and internalization (34.1 ± 4.9%IA/10^6^ at 48 h) in LNCaP cells. In vivo PET analysis showed higher tumor uptake (23.8 ± 11.5%IA/cc at 4 h) in [^64^Cu]Cu-DOTHA_2_-PSMA group, which was significantly reduced in blocking group (*p* < 0.05). Biodistribution studies revealed low kidney accumulation (< 15.8 ± 7.0%ID/g), while liver uptake was relatively higher (30.2 ± 4.2%ID/g at 4 h). Therapeutically, [^64^Cu]Cu-DOTHA_2_-PSMA extended survival in treated mice more effectively than the [^nat^Cu]Cu-DOTHA_2_-PSMA control group (*p* < 0.001) and slightly better than [^177^Lu]Lu-PSMA-617 (*p* = 0.09) with manageable toxicity and minimal off-target effect (Fig. 8). This approach offers a complementary theranostic option with a unique mechanism involving both β-particles and high-LET Auger electrons, enabling more personalized treatment [49]. While conventional β-emitters like lutetium-177 are clinically established; their relatively low LET and longer tissue penetration can limit efficacy, particularly in micrometastatic or heterogeneous tumor environments as much of the emitted energy is dissipated over millimeter distances instead of being deposited within targeted tumor cells. This limitation dictates the growing interest in alternative approaches such as α-particle radiotherapy to improve therapeutic outcomes.

### Alpha Emitter Labeled PSMA-Targeted Agents

PSMA-targeting ligands labeled with α-emitting radionuclides such as actinium-225, astatine-211, lead-212, radium-223 and thorium-227 have gained increasing attention in preclinical due to their high LET and potent cytotoxic effects against tumor cells [70]. Among these, [^225^Ac]Ac-PSMA-617 has been the most extensively studied, showing remarkable efficacy in patients with mCRPC, including those resistant to prior β-therapy [71]. However, limitations such as off-target salivary gland toxicity and limited global availability have shifted the focus towards alternative α-emitters. ^212^Pb-labelled compounds such as [^212^Pb]Pb-CA012 [50] and [^212^Pb]Pb-NG001 [51, 52] have emerged as promising candidates with potent tumor control and favorable safety profiles in preclinical models. The ongoing research indicates that these agents are now moving from preclinical development into clinical investigation. Parallel efforts were made to develop ^223^Ra-tagged PSMA-targeted agents using macropa-chelator, but it faced significant challenges, including in vivo instability and poor tumor delivery [60]. Comparative studies with high LET α-therapy, [^225^Ac]Ac-L1 and [^213^Bi]Bi-L1, confirmed its superior efficacy over β-therapy, [^177^Lu]Lu-L1, in both xenograft and micrometastatic prostate cancer models. In the micrometastatic model, single-dose [^177^Lu]Lu-L1 (37 MBq) showed no survival benefit (47 vs 48 days in control), whereas single dose [^225^Ac]Ac-L1 (37 and 74 kBq) significantly prolonged median survival (56 and 79 days). Fractionated [^225^Ac]Ac-L1 (9.3 kBq × 6) achieved the greatest median survival (72 vs 44 days in controls) and effectively delayed tumor growth without inducing off-target toxicity [53].

**Fig. 9:**
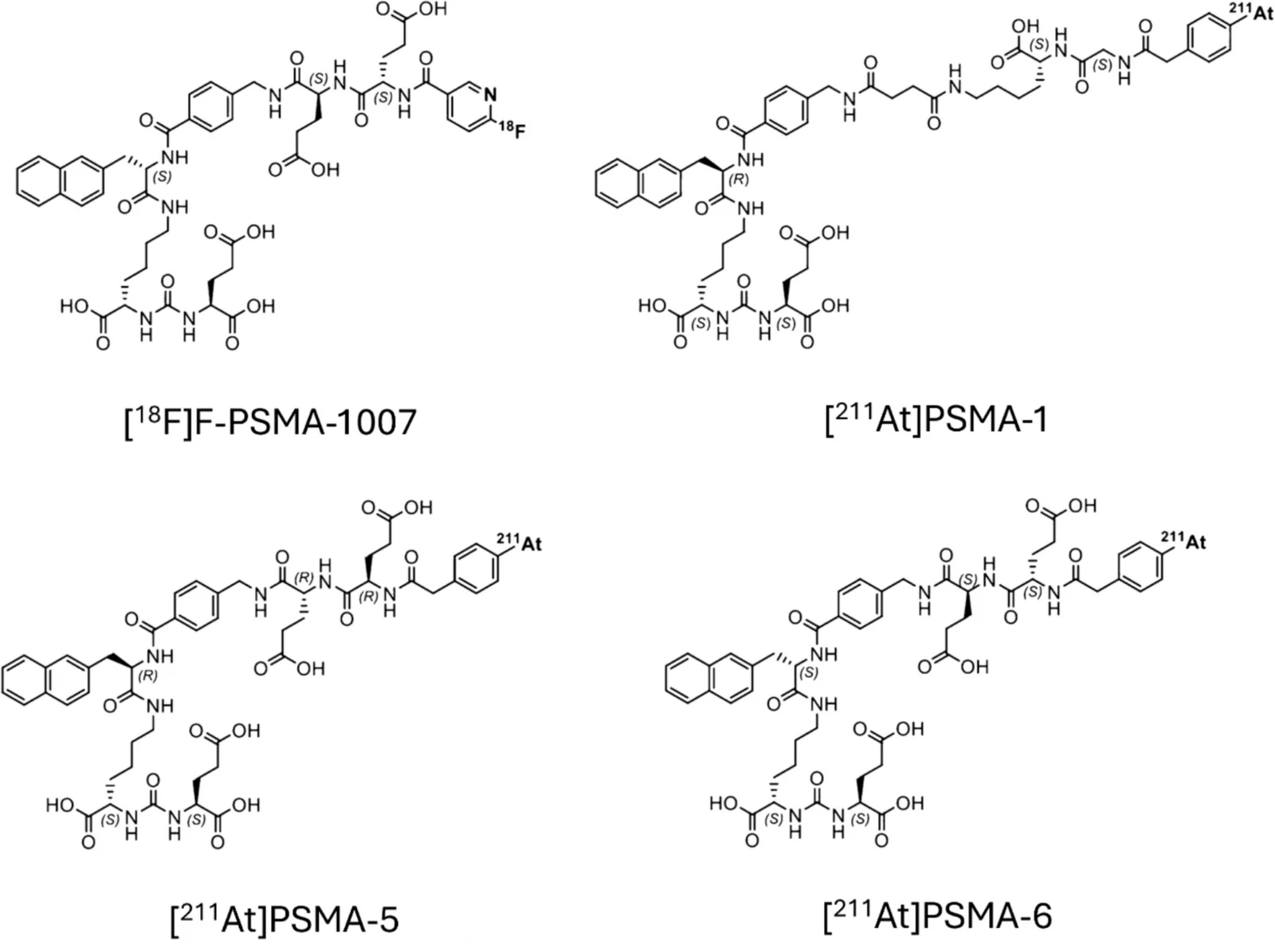
Chemical structures of [^211^At]PSMA-1, [^211^At]PSMA-5, and [^211^At]PSMA-6 derived from the [^18^F]F-PSMA-1007. Adapted from Watabe, T, et al. Targeted α-therapy using astatine (^211^At)-labeled PSMA1, 5, and 6: a preclinical evaluation as a novel compound. Eur J Nucl Med Mol Imaging. 2023;50:849–58. Copyright © 2023 by the authors [55]

**Fig. 10:**
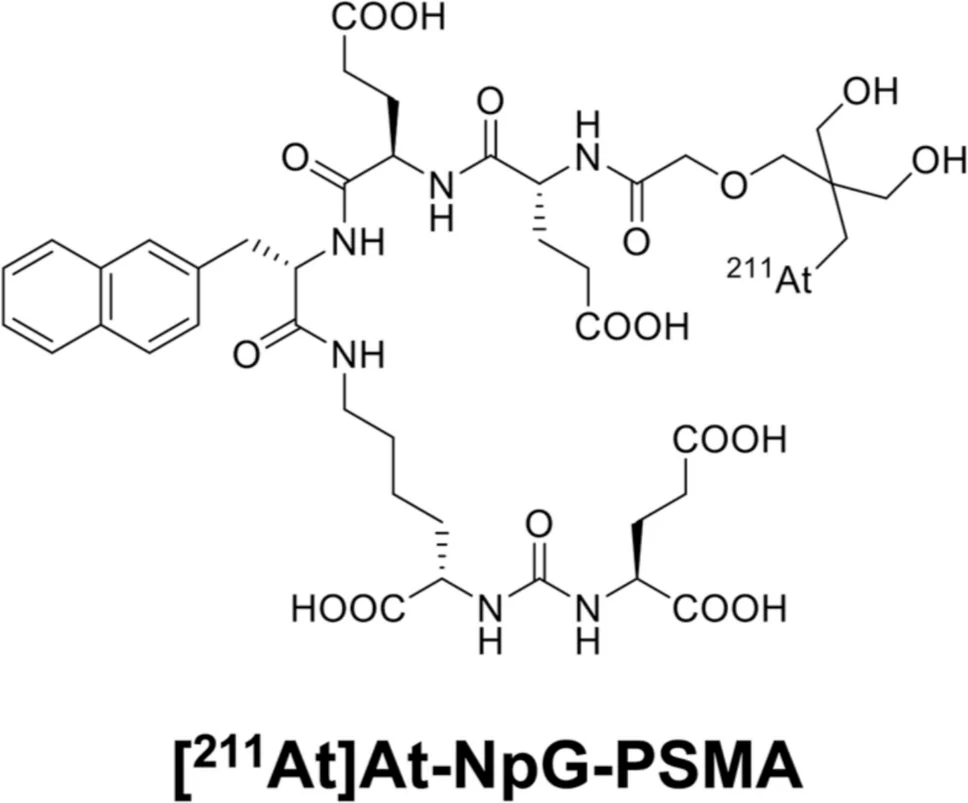
Chemical structures of [^211^At]At-NpG-PSMA. Adapted from Yaginuma K, et al. Novel astatine (^211^At)-labelled prostate-specific membrane antigen ligand with a neopentyl-glycol structure: evaluation of stability, efficacy, and safety using a prostate cancer xenograft model. Eur J Nucl Med Mol Imaging. 2025;52:469–81. Copyright © 2025 by the authors [56]

Astatine-211 is emerging as a promising radionuclide gaining attention due to its short half-life (7.2 h) and reliable cyclotron-based production. A novel ^211^At-labelled PSMA-targeting radiotracer ([^211^At]At-3-Lu) was designed with the goal of an optimized pharmacokinetic profile to enhance therapeutic efficacy. In PC3-PIP xenograft models, [^211^At]At-3-Lu showed dose-dependent tumor growth suppression with favorable biodistribution characteristics. Unlike earlier ^211^At-labelled compounds such as DCABzL, HS-549, GV-620 and GV-904, [^211^At]At-3-Lu demonstrated comparatively little deastatination and exhibited minimal uptake in non-target organs such as salivary glands and kidneys confirmed by stable blood parameters, normal tissue chemistries, and histology over long-term observation [54]. To further refine targeting specificity and therapeutic index, additional ^211^At-labelled PSMA ligands such as PSMA-1, PSMA-5, and PSMA-6 were synthesized using the [^18^F]F-PSMA-1007 scaffold (Fig. 9). An aryl boronic acid was introduced for ^211^At-labeling of PSMA precursors, replacing the N,N,N-trimethyl-2-pyridinaminium group used for ^18^F labeling. The three PSMA analogs differ in side-chain residues: Gly-Lys (PSMA-1), (R)-Glu-(R)-Glu (PSMA-5), and (S)-Glu-(S)-Glu (PSMA-6). Tumor retention of [^211^At]PSMA-5 was markedly higher than that of [^211^At]PSMA-1 and [^211^At]PSMA-6 (30.6 ± 17.8, 12.4 ± 4.8, and 19.1 ± 4.5%ID/g) at 3 h and (40.7 ± 2.6, 8.7 ± 3.5, and 18.1 ± 2.2%ID/g) 24 h respectively, with minimal deastatination (≤ 1% free astatide in urine at 3 h). [^211^At]PSMA-5 exhibited strongest tumor growth suppression, reducing tumor volume by 72% at 4 weeks post-treatment, compared with [^211^At]PSMA-1, which reduced tumor volume by 45%. [^211^At]PSMA-5 also showed minimal off-target toxicity, including xerostomia and nephrotoxicity, supporting its potential for clinical translation [55]. More recently, neopentyl-glycol-PSMA (NpG-PSMA), another [^18^F]F-PSMA-1007 analogue, was developed with a NPG moiety that stabilizes ^211^At-labeling and enhances its resistance to deastatination. [^211^At]At-NpG-PSMA (Fig. 10) was evaluated for its tumor-targeting performance, pharmacokinetics, safety profile, and in vivo stability. In comparison with agents like [^211^At]At-3-Lu which showed tumor uptake of 30, 17, and 10%ID/g at 1, 4, and 24 h, [^211^At]At-NpG-PSMA achieved high and sustained tumor accumulation of 48, 42, and 41%ID/g at 1, 3, and 24 h. Negligible accumulation in thyroid, salivary glands, and stomach at 3 h (0.28, 0.88, and 0.71%ID/g respectively), reflecting low deastatination of [^211^At]At-NpG-PSMA, compared with [^211^At]PSMA-5 (11, 11, and 5%ID/g respectively). Renal toxicity, a common dose-limiting factor in α-therapy, was observed to be minimal and reversible at therapeutically relevant doses, and no significant hematologic toxicity was reported [56].

**Fig. 11:**
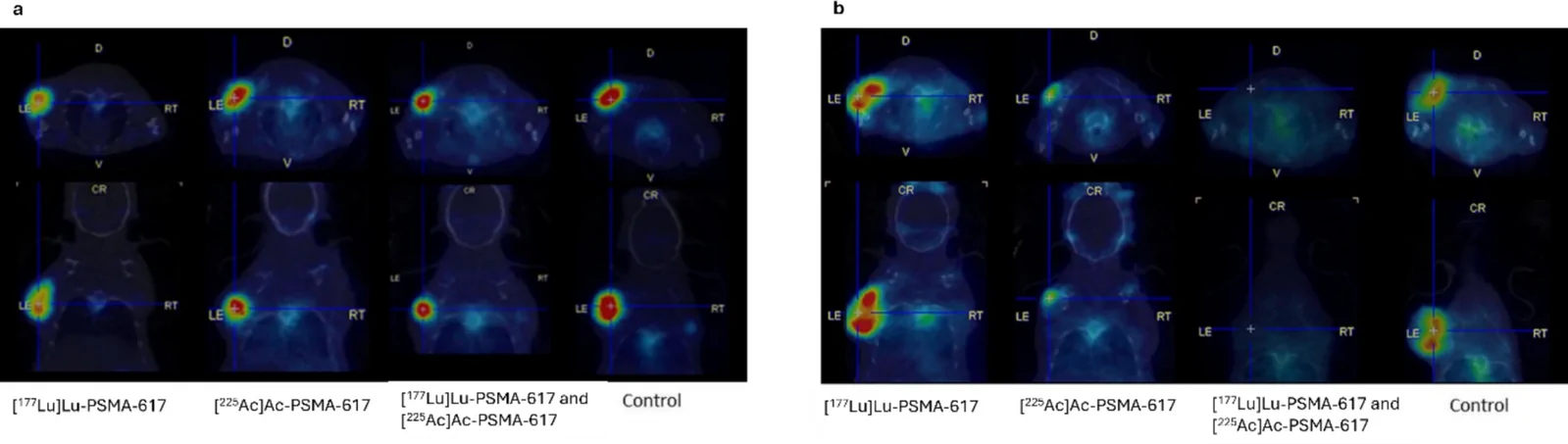
Representative [^18^F]AlF-PSMA-11 PET image of each treatment group 7 days (**a**) and 30 days (**b**) after treatment. Adapted from Savio E, et al., Nucl Med Biol. 2025;146–147:109,032. © 2025, with permission from Elsevier. Licensed under CC BY-NC-ND 4.0 [72]

The focus of RLT research is gradually shifting toward cocktail therapies that combine α and β-emitters. A recent study evaluated the therapeutic potential of [^225^Ac]Ac-PSMA-617 and investigated the in vivo effects of its combination with the [^177^Lu]Lu-PSMA-617 in preclinical models of prostate cancer. A four-arm study in xenografted nude mice assessed the therapeutic effects of monotherapy and combination regimens. In control group, tumor detectability remained 100%, but survival decreased from 56% at 3rd week to 0% in 4th week. Monotherapy groups, [^177^Lu]Lu-PSMA-617 and [^225^Ac]Ac-PSMA-617 maintained 100% survival until 3rd week, then decreased to 33% and 50% by 8th week, with tumor detectability declining to 78% and 60%, respectively. Mice receiving the combined [^225^Ac]Ac-PSMA-617/[^177^Lu]Lu-PSMA-617 treatment (Fig. 11) showed the most favorable outcomes, including delayed tumor growth, 90% survival at 8th week and complete tumor regression in 40% of cases, as monitored by PET/CT and caliper measurements [72].

**Fig. 12:**
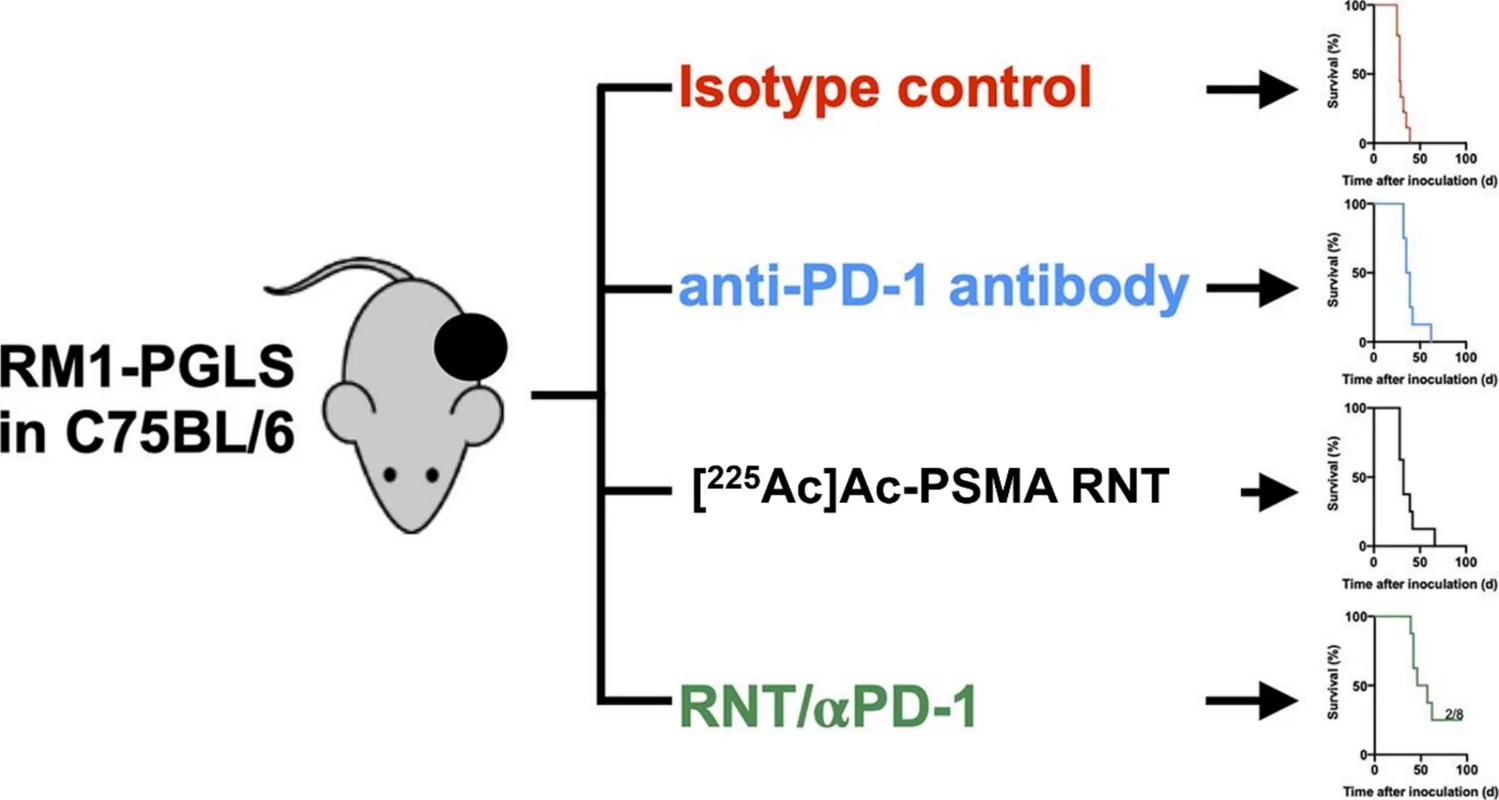
Comparison of the survival (%) of C57BL/6 mice bearing RM1-PGLS tumors in different treatment groups. This research was originally published in JNM. Czernin J, et al. Immune-Checkpoint Blockade Enhances.^225^Ac-PSMA617 Efficacy in a Mouse Model of Prostate Cancer. J Nucl Med. 2021;62:228–31. © SNMMI [74]

Researchers are also exploring other cocktail therapies to combine PSMA targeted RLT with immunotherapy aims to amplify antitumor efficacy by exploiting various synergistic mechanisms. First, local cytotoxicity and subsequent immunogenic cell death is induced by PSMA-targeted agents (e.g., therapeutic radionuclides) that releases tumor antigens and damage-associated molecular patterns (DAMPs). This process effectively transforms immunologically "cold" tumors into "hot" phenotypes through enhanced dendritic cell maturation and antigen presentation and making them sensitize to immune checkpoint inhibitors. Second, PSMA ligands can be used to deliver immune-stimulatory agents, such as 4-1BB agonists or cytokines, ensuring their presence directly within the tumor microenvironment to enhance local immune activation. Third, radionuclide-induced DNA damage leads to antigen release that, when paired with anti-PD-1 therapy, generates tumor-specific memory T-cells to reduce tumor recurrence [73]. A similar investigation was done by using the combination of RLT ([^225^Ac]Ac-PSMA-617) and immune checkpoint blockade using an anti-PD-1 antibody in C57BL/6-mice bearing syngeneic RM1-PGLS tumors. The combination of [^225^Ac]Ac-PSMA-617 and an anti-PD-1 antibody significantly extended median survival to 51.5 days, compared to 37 days for anti-PD-1 alone and 32 days for RLT alone (Fig. 12). This result was statistically superior to either monotherapy (P ≤ 0.0251), providing strong preclinical evidence of combining these modalities to achieve synergistic therapeutic outcomes [74]. The promising preclinical results from this combination therapy strategy suggest a potential advancement in overcoming resistance in prostate cancer treatment. Furthermore, the development of theranostic agents compatible with novel radionuclides like lead-212, and astatine-211 supports the emergence of more personalized and scalable therapeutic paradigms.

### Auger Emitter Labeled PSMA-Targeted Agents

**Fig. 13:**
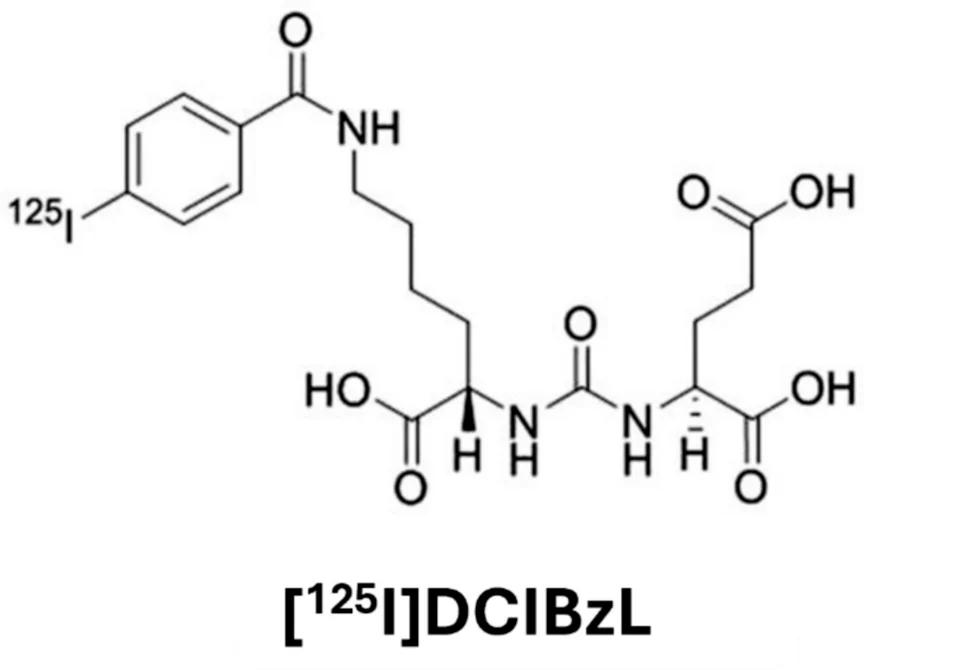
Chemical structure of [^125^I]DCIBzL. This research was originally published in JNM. Kiess AP, et al. Auger Radiopharmaceutical Therapy Targeting Prostate-Specific Membrane Antigen. J Nucl Med. 2015;56:1401–7. © SNMMI [57]

Auger emitter-labeled PSMA-targeted agents have been developed to address the need for highly localized, potent therapies against micrometastatic prostate cancer. Unlike β and α-emitters, which have longer tissue penetration and can cause off-target toxicity, Auger electrons emit extremely short-range, high-LET radiation ideal for precise delivery to intracellular and preferably nuclear targets to maximize cytotoxicity. Successful efforts have designed small-molecule PSMA ligands such as [^125^I]DCIBzL (2-[3-[1-carboxy-5-(4-[^125^I]iodobenzoylamino)-pentyl]-ureido]-pentanedioic acid) (Fig. 13) with enhanced cellular internalization and nuclear proximity, optimizing the spatial localization of decay events. Kiess et al. developed a highly specific small-molecule PSMA-targeting agent, [^125^I]DCIBzL, to deliver iodine-125 selectively to prostate cancer cells. In vitro, treatment with [^125^I]DCIBzL induced significant DNA damage and reduced clonogenic survival in PSMA-positive PC3 PIP cells, but not in PSMA-negative PC3 flu cells. Confocal microscopy confirmed the compound localized to the perinuclear region and plasma membrane. In vivo, nude mice bearing PSMA-positive PC3 PIP xenografts treated intravenously with 111 MBq, [^125^I]DCIBzL showed strong tumor growth suppression, with only 1 of 6 mice reaching 5 × the initial tumor volume by day 60, compared with < 15 days in PSMA-negative tumors and all control groups (*p* = 0.002). The sustained tumor responses were likely due to the highest PSMA-binding affinity (K_i_ = 0.01 nM) of [^125^I]DCIBzL, ensuring prolonged tumor retention, and the long half-life (59 days) of iodine-125, providing continuous radiation exposure and effective DNA damage. No weight loss or acute renal and hematologic toxicity was observed at the administered dose. These findings demonstrated the therapeutic potential of PSMA-targeted Auger radiotherapy for selective eradication of prostate cancer micrometastases [57].

Based on this investigation, Shen et al. advanced the research by evaluating therapeutic potential of [^125^I]DCIBzL and its long-term safety in a micrometastatic prostate cancer model, more closely reflecting the clinical context of early metastatic disease using PSMA-positive PC3-ML cells in NSG mice. Treatment with ≥ 18.5 MBq of [^125^I]DCIBzL significantly delayed metastatic progression and prolonged survival from 6 weeks in controls to 11 weeks in the treatment group with no appreciable long-term toxicity observed over 12 months. Compared to α-emitters like [^211^At]At-PSMA-6, which exhibited dose-limiting nephrotoxicity with a maximum tolerated dose (MTD) of 37 kBq in CD1 mice, the MTD of [^125^I]DCIBzL exceeded 111 MBq. Dosimetry modeling confirmed selective tumor targeting and low renal exposure, supporting the potential of Auger-based PSMA-RLT as a safe and effective strategy [39]. Expanding upon prior efforts with iodine-based Auger emitters and in line with growing interest in PSMA-targeted Auger radiolands, Baun et al. investigated [^58m^Co]Co-DOTA-PSMA-617, for evaluating its therapeutic efficacy and safety in preclinical models. In vitro studies confirmed PSMA-specific uptake, significant nuclear accumulation, and dose-dependent reductions in cell viability and clonogenic survival (*p* < 0.05). In-vivo, the compound exhibited strong tumor targeting; two intravenous doses of 144 ± 9 MBq of [^58m^Co]Co-DOTA-PSMA-617 (at 7 and 14 day) significantly prolonged median survival compared to controls (23 days vs. 11 days, *p* = 0.0014). No weight loss, hematologic or organ toxicity was observed and salivary glands and bone marrow remained unaffected, supporting the potential of cobalt-based Auger therapy as an effective and safe approach for prostate cancer [58].

Recent investigations by Bobba et al. explored an Auger-based theranostic agent, [^134^Ce]Ce/[^134^La]La-PSMA-617, for both PET imaging and Auger electron therapy in prostate cancer models. The agent exhibited high radiochemical purity, excellent serum stability, and strong PSMA-specific binding and internalization in vitro. In vivo PET imaging and biodistribution studies demonstrated selective tumor uptake and rapid clearance from non-target tissues. A single-dose administration of [^134^Ce]Ce-PSMA-617 significantly prolonged survival in PSMA-positive tumor-bearing mice in a dose-dependent manner, with median survival extended from 33 to 80 days. These results suggest the promise of [^134^Ce]Ce-PSMA-617 as a dual-function agent, combining diagnostic imaging with effective Auger-mediated radiotherapy for micrometastatic prostate cancer [59]. Despite encouraging preclinical outcomes, PSMA-targeted small-molecules labeled with Auger emitters have not yet progressed to clinical approval and remain in early stages of development.

## Conclusion

The emergence of small-molecule PSMA-targeted radioligands has transformed the management of advanced prostate cancer, paving the way for new therapeutic approaches. These low molecular weight urea-based compounds bind PSMA with high affinity, allowing rapid tumor penetration, faster systemic clearance, and efficient delivery of radionuclides. The clinical success of PSMA small molecule labeled with β-emitter, [^177^Lu]Lu-PSMA-617, has demonstrated significant survival benefits by delivering moderate-energy β-particles enabling effective treatment of both primary tumors and metastatic lesions. However, challenges such as acquired resistance and off-target toxicities, including xerostomia and myelosuppression, highlight the need for continued innovation.

To overcome these obstacles, preclinical research has explored advanced strategies to enhance therapeutic efficacy and safety. Rational PSMA ligand optimization through ABM and linker modifications have been attempted and the optimized one labeled with β-emitters improved pharmacokinetics, increasing tumor-to-organ dose ratios and prolonging anti-tumor effects in prostate cancer models. However, these efforts still come at the cost of increased renal retention, achieving the optimal balance between high tumor accumulation and minimal off-target exposure remains a central challenge in PSMA-targeted radioligand design. Moreover, the use of β-emitters, characterized by relatively low LET and long tissue penetration range, may limit their therapeutic efficacy in micrometastatic disease or within heterogeneous tumor microenvironments. PSMA ligands labeled with high-energy α-emitters have been developed, offering potent cytotoxic effects against micrometastatic prostate cancer. In addition, Auger electron emitters have emerged as promising candidates, providing highly localized cytotoxicity while minimizing collateral damage to surrounding healthy tissues.

PSMA-targeted RLT is moving toward personalized and combination approaches. The design of innovative combination therapies, which may involve pairing different classes of radionuclides or integrating radioligand therapy with other treatment modalities like immunotherapy, is a highly promising frontier. Continued progress in the development of novel theranostic agents and the establishment of sustainable, scalable isotope production methods are essential to unlock the full potential of PSMA-targeted therapies for broader clinical application.

## Data Availability

Not applicable.

## References

[1] James ND, Tannock I, N’Dow J, Feng F, Gillessen S, Ali SA, et al. The Lancet commission on prostate cancer: planning for the surge in cases. Lancet. 2024;403:1683–722. 10.1016/S0140-6736(24)00651-2 PMC7617369 38583453

[2] Bray F, Laversanne M, Sung H, Ferlay J, Siegel RL, Soerjomataram I, et al. Global cancer statistics 2022: GLOBOCAN estimates of incidence and mortality worldwide for 36 cancers in 185 countries. CA Cancer J Clin. 2024;74:229–63. 10.3322/caac.21834 38572751

[3] Varaprasad GL, Gupta VK, Prasad K, Kim E, Tej MB, Mohanty P, et al. Recent advances and future perspectives in the therapeutics of prostate cancer. Exp Hematol Oncol. 2023;12:80. 10.1186/s40164-023-00444-9 PMC10517568 37740236

[4] Zhong J, Jang A, Garcia J, Avril N, Li Q, Wojtylak P, et al. Advances in prostate cancer treatment: radionuclide therapy for prostate cancer. Adv Cancer Res. 2024;164:311–58. 10.1016/bs.acr.2024.07.004 39306369

[5] Watson PA, Arora VK, Sawyers CL. Emerging mechanisms of resistance to androgen receptor inhibitors in prostate cancer. Nat Rev Cancer. 2015;15:701–11. 10.1038/nrc4016 PMC4771416 26563462

[6] Karantanos T, Corn PG, Thompson TC. Prostate cancer progression after androgen deprivation therapy: mechanisms of castrate resistance and novel therapeutic approaches. Oncogene. 2013;32:5501–11. 10.1038/onc.2013.206 PMC3908870 23752182

[7] Jang A, Kendi AT, Sartor O. Status of PSMA-targeted radioligand therapy in prostate cancer: current data and future trials. Ther Adv Med Oncol. 2023;15:17588359231157632. 10.1177/17588359231157632 PMC9989419 36895851

[8] Wolf P, Gierschner D, Bühler P, Wetterauer U, Elsässer-Beile U. A recombinant PSMA-specific single-chain immunotoxin has potent and selective toxicity against prostate cancer cells. Cancer Immunol Immunother. 2006;55:1367–73. 10.1007/s00262-006-0131-0 PMC11030148 16547705

[9] Ross JS, Sheehan CE, Fisher HAG, Kaufman RP Jr, Kaur P, Gray K, et al. Correlation of primary tumor prostate-specific membrane antigen expression with disease recurrence in prostate cancer. Clin Cancer Res. 2003;9:6357–62. 14695135

[10] Heynickx N, Herrmann K, Vermeulen K, Baatout S, Aerts A. The salivary glands as a dose limiting organ of PSMA- targeted radionuclide therapy: a review of the lessons learnt so far. Nucl Med Biol. 2021;98–9:30–9. 10.1016/j.nucmedbio.2021.04.003 34020337

[11] Ceci F, Fanti S. PSMA-PET/CT imaging in prostate cancer: why and when. Clin Transl Imaging. 2019;7:377–9.

[12] Miyahira AK, Pienta KJ, Morris MJ, Bander NH, Baum RP, Fendler WP, et al. Meeting report from the prostate cancer foundation PSMA-directed radionuclide scientific working group. Prostate. 2018;78:775–89. 10.1002/pros.23642 29717499

[13] Barrio M, Fendler WP, Czernin J, Herrmann K. Prostate specific membrane antigen (PSMA) ligands for diagnosis and therapy of prostate cancer. Expert Rev Mol Diagn. 2016;16:1177–88. 10.1080/14737159.2016.1243057 27679869

[14] Eiber M, Fendler WP, Rowe SP, Calais J, Hofman MS, Maurer T, et al. Prostate-specific membrane antigen ligands for imaging and therapy. J Nucl Med. 2017;58:67S-76S. 10.2967/jnumed.116.186767 28864615

[15] Neagu M, Constantin C, Hinescu ME, Bleotu PG, Popovici M-G, Zai M-I, et al. Radionuclides landscape in prostate cancer theranostics. Int J Mol Sci. 2025;26:6751. 10.3390/ijms26146751 PMC12296053 40724999

[16] El Fakiri M, Geis NM, Ayada N, Eder M, Eder A-C. PSMA-targeting radiopharmaceuticals for prostate cancer therapy: recent developments and future perspectives. Cancers. 2021;13:3967. 10.3390/cancers13163967 PMC8393521 34439121

[17] Ayzman A, Pachynski RK, Reimers MA. PSMA-based therapies and novel therapies in advanced prostate cancer: the now and the future. Curr Treat Options Oncol. 2025;26:375–84. 10.1007/s11864-025-01317-5 PMC12055665 40266437

[18] Uemura M, Watabe T, Hoshi S, Tanji R, Yaginuma K, Kojima Y. The current status of prostate cancer treatment and PSMA theranostics. Ther Adv Med Oncol. 2023;15:17588359231182292. 10.1177/17588359231182293 PMC10328176 37424944

[19] Puik JR, Le C, Kazemier G, Oprea-Lager DE, Swijnenburg R-J, Giovannetti E, et al. Prostate-specific membrane antigen as target for vasculature-directed therapeutic strategies in solid tumors. Crit Rev Oncol Hematol. 2025;205:104556. 10.1016/j.critrevonc.2024.104556 39551117

[20] He Y, Xu W, Xiao Y-T, Huang H, Gu D, Ren S. Targeting signaling pathways in prostate cancer: mechanisms and clinical trials. Signal Transduct Target Ther. 2022;7:198. 10.1038/s41392-022-01042-7 PMC9232569 35750683

[21] Abusalem M, Martiniova L, Soebianto S, DePalatis L, Ravizzini G. Current status of radiolabeled monoclonal antibodies targeting PSMA for imaging and therapy. Cancers. 2023;15:4537. 10.3390/cancers15184537 PMC10526399 37760506

[22] Khalid U, Vi C, Henri J, Macdonald J, Eu P, Mandarano G, et al. Radiolabelled aptamers for theranostic treatment of cancer. Pharmaceuticals. 2018;12:2. 10.3390/ph12010002 PMC6469178 30586898

[23] Donin NM, Reiter RE. Why targeting PSMA is a game changer in the management of prostate cancer. J Nucl Med. 2018;59:177–82. 10.2967/jnumed.117.191874 PMC6910619 28986509

[24] Sallam M, Nguyen N-T, Sainsbury F, Kimizuka N, Muyldermans S, Benešová-Schäfer M. PSMA-targeted radiotheranostics in modern nuclear medicine: then, now, and what of the future? Theranostics. 2024;14:3043–79. 10.7150/thno.92612 PMC11155394 38855174

[25] Haupt F, Dijkstra L, Alberts I, Sachpekidis C, Fech V, Boxler S, et al. ^68^Ga-PSMA-11 PET/CT in patients with recurrent prostate cancer-a modified protocol compared with the common protocol. Eur J Nucl Med Mol Imaging. 2020;47:624–31. 10.1007/s00259-019-04548-5 31673789

[26] Rowe SP, Gage KL, Faraj SF, Macura KJ, Cornish TC, Gonzalez-Roibon N, et al. 1⁸F-DCFBC PET/CT for PSMA-based detection and characterization of primary prostate cancer. J Nucl Med. 2015;56:1003–10. 10.2967/jnumed.115.154336 PMC4659400 26069305

[27] Rowe SP, Macura KJ, Mena E, Blackford AL, Nadal R, Antonarakis ES, et al. PSMA-based [^18^F]DCFPyL PET/CT is superior to conventional imaging for lesion detection in patients with metastatic prostate cancer. Mol Imaging Biol. 2016;18:411–9. 10.1007/s11307-016-0957-6 PMC5261857 27080322

[28] Barrett JA, Coleman RE, Goldsmith SJ, Vallabhajosula S, Petry NA, Cho S, et al. First-in-man evaluation of 2 high-affinity PSMA-avid small molecules for imaging prostate cancer. J Nucl Med. 2013;54:380–7. 10.2967/jnumed.112.111203 23303962

[29] Gourni E, Henriksen G. Metal-based PSMA radioligands. Molecules. 2017;22:523. 10.3390/molecules22040523 PMC6154343 28338640

[30] Debnath S, Zhou N, McLaughlin M, Rice S, Pillai AK, Hao G, et al. PSMA-targeting imaging and theranostic agents-current status and future perspective. Int J Mol Sci. 2022;23:1158. 10.3390/ijms23031158 PMC8835702 35163083

[31] Okarvi SM. Recent developments of prostate-specific membrane antigen (PSMA)-specific radiopharmaceuticals for precise imaging and therapy of prostate cancer: an overview. Clin Transl Imaging. 2019;7:189–208.

[32] Benešová M, Schäfer M, Bauder-Wüst U, Afshar-Oromieh A, Kratochwil C, Mier W, et al. Preclinical evaluation of a tailor-made DOTA-conjugated PSMA inhibitor with optimized linker moiety for imaging and endoradiotherapy of prostate cancer. J Nucl Med. 2015;56:914–20. 10.2967/jnumed.114.147413 25883127

[33] Eder A-C, Schäfer M, Schmidt J, Bauder-Wüst U, Roscher M, Leotta K, et al. Rational linker design to accelerate excretion and reduce background uptake of peptidomimetic PSMA-targeting hybrid molecules. J Nucl Med. 2021;62:1461–7. 10.2967/jnumed.120.248443 PMC8724895 33741642

[34] Ferro-Flores G, Azorín-Vega E, Ocampo-García B, Luna-Gutiérrez M, Cruz-Nova P, Meléndez-Alafort L. Effects of targeted radionuclide therapy on cancer cells beyond the ablative radiation dose. Int J Mol Sci. 2025;26:6968. 10.3390/ijms26146968 PMC12296037 40725216

[35] Salih S, Alkatheeri A, Alomaim W, Elliyanti A. Radiopharmaceutical treatments for cancer therapy, radionuclides characteristics, applications, and challenges. Molecules. 2022;27:5231. 10.3390/molecules27165231 PMC9415873 36014472

[36] Parsi M, Desai MH, Desai D, Singhal S, Khandwala PM, Potdar RR. PSMA: a game changer in the diagnosis and treatment of advanced prostate cancer. Med Oncol. 2021;38:89. 10.1007/s12032-021-01537-3 34181109

[37] Nelson BJB, Andersson JD, Wuest F. Targeted alpha therapy: progress in radionuclide production, radiochemistry, and applications. Pharmaceutics. 2021;13:49. 10.3390/pharmaceutics13010049 PMC7824049 33396374

[38] Arbuznikova D, Eder M, Grosu AL, Meyer PT, Gratzke C, Zamboglou C, et al. Towards improving the efficacy of PSMA-targeting radionuclide therapy for late-stage prostate cancer-combination strategies. Curr Oncol Rep. 2023;25:1363–74. 10.1007/s11912-023-01458-6 PMC10640479 37861915

[39] Sathekge MM, Bruchertseifer F, Vorster M, Morgenstern A, Lawal IO. Global experience with PSMA-based alpha therapy in prostate cancer. Eur J Nucl Med Mol Imaging. 2021;49:30–46. 10.1007/s00259-021-05434-9 PMC8712297 34173838

[40] Lacerda S, de Kruijff RM, Djanashvili K. The advancement of targeted alpha therapy and the role of click chemistry therein. Molecules. 2025;30:1296. 10.3390/molecules30061296 PMC11944744 40142070

[41] Czerwińska M, Bilewicz A, Kruszewski M, Wegierek-Ciuk A, Lankoff A. Targeted radionuclide therapy of prostate cancer-from basic research to clinical perspectives. Molecules. 2020;25:1743. 10.3390/molecules25071743 PMC7181060 32290196

[42] Kuo H-T, Merkens H, Zhang Z, Uribe CF, Lau J, Zhang C, et al. Enhancing treatment efficacy of ^177^Lu-PSMA-617 with the conjugation of an albumin-binding motif: preclinical dosimetry and endoradiotherapy studies. Mol Pharm. 2018;15:5183–91. 10.1021/acs.molpharmaceut.8b00720 30251544

[43] Kuo HT, Lin KS, Zhang Z, Uribe CF, Merkens H, Zhang C, et al. ^177^Lu-labeled albumin-binder-conjugated PSMA-targeting agents with extremely high tumor uptake and enhanced tumor-to-kidney absorbed dose ratio. J Nucl Med. 2021;62:521–7. 10.2967/jnumed.120.250738 PMC8049373 32859704

[44] Benešová M, Umbricht CA, Schibli R, Müller C. Albumin-binding PSMA ligands: optimization of the tissue distribution profile. Mol Pharm. 2018;15:934–46. 10.1021/acs.molpharmaceut.7b00877 29400475

[45] Umbricht CA, Benesova M, Schibli R, Müller C. Preclinical development of novel PSMA-targeting radioligands: modulation of albumin-binding properties to improve prostate cancer therapy. Mol Pharm. 2018;15:2297–306. 10.1021/acs.molpharmaceut.8b00152 29684274

[46] Kelly J, Amor-Coarasa A, Ponnala S, Nikolopoulou A, Williams C Jr, Schlyer D, et al. Trifunctional PSMA-targeting constructs for prostate cancer with unprecedented localization to LNCaP tumors. Eur J Nucl Med Mol Imaging. 2018;45:1841–51. 10.1007/s00259-018-4004-5 29623376

[47] Kelly JM, Amor-Coarasa A, Ponnala S, Nikolopoulou A, Williams C, DiMagno SG, et al. Albumin-binding PSMA ligands: implications for expanding the therapeutic window. J Nucl Med. 2019;60:656–63. 10.2967/jnumed.118.221150 30552199

[48] Kelly JM, Amor-Coarasa A, Nikolopoulou A, Wüstemann T, Barelli P, Kim D, et al. Dual-target binding ligands with modulated pharmacokinetics for endoradiotherapy of prostate cancer. J Nucl Med. 2017;58:1442–9. 10.2967/jnumed.116.188722 28450562

[49] Milot M-C, Benesty OB, Dumulon-Perreault V, Ait-Mohand S, Geha S, Richard P, et al. The journey to a novel theranostic for prostate cancer: the preclinical study of ^64^Cu-DOTHA_2_-PSMA. J Nucl Med. 2022;63:4025.

[50] Dos Santos JC, Schäfer M, Bauder-Wüst U, Lehnert W, Leotta K, Morgenstern A, et al. Development and dosimetry of ^203^Pb/^212^Pb-labelled PSMA ligands: bringing “the lead” into PSMA-targeted alpha therapy? Eur J Nucl Med Mol Imaging. 2019;46:1081–91. 10.1007/s00259-018-4220-z PMC6451745 30603987

[51] Stenberg VY, Larsen RH, Ma LW, Peng Q, Juzenas P, Bruland ØS, et al. Evaluation of the PSMA-binding ligand 212Pb-NG001 in multicellular tumour spheroid and mouse models of prostate cancer. Int J Mol Sci. 2021;22:4815. 10.3390/ijms22094815 PMC8124365 34062920

[52] Stenberg VY, Juzeniene A, Chen Q, Yang X, Bruland ØS, Larsen RH. Preparation of the alpha-emitting prostate-specific membrane antigen targeted radioligand [^212^Pb]Pb-NG001 for prostate cancer. J Labelled Comp Radiopharm. 2020;63:129–43. 10.1002/jlcr.3825 31919866

[53] Banerjee SR, Lisok A, Minn I, Josefsson A, Kumar V, Brummet M, et al. Preclinical evaluation of ^213^Bi- and ^225^Ac-labeled low-molecular-weight compounds for radiopharmaceutical therapy of prostate cancer. J Nucl Med. 2021;62:980–8. 10.2967/jnumed.120.256388 PMC8882883 33246975

[54] Mease RC, Kang CM, Kumar V, Banerjee SR, Minn I, Brummet M, et al. An improved ^211^At-labeled agent for PSMA-targeted α-therapy. J Nucl Med. 2022;63:259–67. 10.2967/jnumed.121.262098 PMC8805774 34088772

[55] Watabe T, Kaneda-Nakashima K, Shirakami Y, Kadonaga Y, Ooe K, Wang Y, et al. Targeted α-therapy using astatine (^211^At)-labeled PSMA1, 5, and 6: a preclinical evaluation as a novel compound. Eur J Nucl Med Mol Imaging. 2023;50:849–58. 10.1007/s00259-022-06016-z PMC9852121 36344651

[56] Yaginuma K, Takahashi K, Hoshi S, Joho T, Shimoyama S, Hasegawa N, et al. Novel astatine (^211^At)-labelled prostate-specific membrane antigen ligand with a neopentyl-glycol structure: evaluation of stability, efficacy, and safety using a prostate cancer xenograft model. Eur J Nucl Med Mol Imaging. 2025;52:469–81. 10.1007/s00259-024-06945-x PMC11732874 39394527

[57] Kiess AP, Minn I, Chen Y, Hobbs R, Sgouros G, Mease RC, et al. Auger radiopharmaceutical therapy targeting prostate-specific membrane antigen. J Nucl Med. 2015;56:1401–7. 10.2967/jnumed.115.155929 PMC4634552 26182968

[58] Baun C, Dam JH, Hildebrandt MG, Ewald JD, Kristensen BW, Gammelsrød VS, et al. Preclinical evaluation of [^58m^Co]Co-DOTA-PSMA-617 for auger electron therapy of prostate cancer. Sci Rep. 2023;13:18837. 10.1038/s41598-023-43429-8 PMC10620164 37914790

[59] Bobba KN, Wadhwa A, Bidkar AP, Raveendran A, Garcia L, Greenland N, et al. Evaluation of ^134^Ce/^134^La-PSMA-617 for PET imaging and auger electron therapy of prostate cancer. J Nucl Med. 2025;66:1252–7. 10.2967/jnumed.125.269751 PMC12320565 40506238

[60] Alati S, Singh R, Pomper MG, Rowe SP, Banerjee SR. Preclinical development in radiopharmaceutical therapy for prostate cancer. Semin Nucl Med. 2023;53:663–86. 10.1053/j.semnuclmed.2023.06.007 37468417

[61] Hennrich U, Eder M. [^177^Lu]Lu-PSMA-617 (pluvicto™): the first FDA-approved radiotherapeutical for treatment of prostate cancer. Pharmaceuticals. 2022;15:1292. 10.3390/ph15101292 PMC9608311 36297404

[62] Konopnicki A, Zaliznyak M, Roy M, Jana B. The therapeutic use of ^177^Lu-PSMA-617 radioligand therapy in prostate cancer treatment: a review of literature and ongoing trials. Discover Oncol. 2024;15:791. 10.1007/s12672-024-01680-z PMC11655789 39692806

[63] Ahmadzadehfar H, Rahbar K, Kürpig S, Bögemann M, Claesener M, Eppard E, et al. Early side effects and first results of radioligand therapy with ^177^Lu-DKFZ-617 PSMA of castrate-resistant metastatic prostate cancer: a two-centre study. EJNMMI Res. 2015;5:36. 10.1186/s13550-015-0114-2 PMC4477007 26099227

[64] Rahbar K, Bode A, Weckesser M, Avramovic N, Claesener M, Stegger L, et al. Radioligand therapy with ^177^Lu-PSMA-617 as a novel therapeutic option in patients with metastatic castration resistant prostate cancer. Clin Nucl Med. 2016;41:522–8. 10.1097/RLU.0000000000001240 27088387

[65] Rahbar K, Ahmadzadehfar H, Kratochwil C, Haberkorn U, Schäfers M, Essler M, et al. German multicenter study investigating ^177^Lu-PSMA-617 radioligand therapy in advanced prostate cancer patients. J Nucl Med. 2017;58:85–90. 10.2967/jnumed.116.183194 27765862

[66] Hofman MS, Violet J, Hicks RJ, Ferdinandus J, Thang SP, Akhurst T. [^177^Lu]-PSMA-617 radionuclide treatment in patients with metastatic castration-resistant prostate cancer (LuPSMA trial): a single-centre, single-arm, phase 2 study. Lancet Oncol. 2018;19:825–33. 10.1016/S1470-2045(18)30198-0 29752180

[67] Hofman MS, Emmett L, Sandhu S, Iravani A, Joshua AM, Goh JC. <article-title update="added">[177Lu]Lu-PSMA-617 versus cabazitaxel in patients with metastatic castration-resistant prostate cancer (TheraP): a randomised, open-label, phase 2 trial. Lancet. 2021;397:797–804. 10.1016/S0140-6736(21)00237-3 33581798

[68] Sartor O, De Bono J, Chi KN, Fizazi K, Herrmann K, Rahbar K, et al. Lutetium-177-PSMA-617 for metastatic castration-resistant prostate cancer. N Engl J Med. 2021;385:1091–103. 10.1056/NEJMoa2107322 PMC8446332 34161051

[69] de Roode KE, Joosten L, Behe M. Towards the magic radioactive bullet: improving targeted radionuclide therapy by reducing the renal retention of radioligands. Pharmaceuticals. 2024;17:256. 10.3390/ph17020256 PMC10892921 38399470

[70] Bidkar AP, Zerefa L, Yadav S, VanBrocklin HF, Flavell RR. Actinium-225 targeted alpha particle therapy for prostate cancer. Theranostics. 2024;14:2969–92. 10.7150/thno.96403 PMC11103494 38773983

[71] Ma J, Zhang Y, Yangqing J, Liu G, Wang J, Zhang C. The efficacy and safety of ^225^Ac-PSMA-617 in metastatic castration-resistant prostate cancer. Front Oncol. 2025;15:1516860. 10.3389/fonc.2025.1516860 PMC11906306 40094004

[72] Savio E, Reyes L, Giglio J, Alfaya L, Falasco G, Urrutia L, et al. Preclinical evaluation of [^225^Ac] ac-PSMA-617 and in vivo effect comparison in combination with [^177^Lu] Lu-PSMA-617 for prostate cancer. Nucl Med Biol. 2025;146–7:109032. 10.1016/j.nucmedbio.2025.109032 40513471

[73] Wang L, Wang Xa, Wu D. PSMA targeted therapy: from molecular mechanisms to clinical breakthroughs in castration-resistant prostate cancer. Eur J Med Chem. 2025;296:117829. 10.1016/j.ejmech.2025.117829 40466338

[74] Czernin J, Current K, Mona CE, Nyiranshuti L, Hikmat F, Radu CG, et al. Immune-checkpoint blockade enhances ^225^Ac-PSMA617 efficacy in a mouse model of prostate cancer. J Nucl Med. 2021;62:228–31. 10.2967/jnumed.120.246041 PMC12079179 32646877

